# Emergence, Evolution, and Biological Characteristics of H10N4 and H10N8 Avian Influenza Viruses in Migratory Wild Birds Detected in Eastern China in 2020

**DOI:** 10.1128/spectrum.00807-22

**Published:** 2022-04-07

**Authors:** Yanwen Wang, Mengjing Wang, Hong Zhang, Conghui Zhao, Yaping Zhang, Guimei He, Guohua Deng, Pengfei Cui, Yubao Li, Wenqiang Liu, Jinyan Shen, Xiaohong Sun, Wenting Wang, Xianying Zeng, Yanbing Li, Dong Chu, Peng Peng, Jing Guo, Hualan Chen, Xuyong Li

**Affiliations:** a College of Agronomy, Liaocheng University, Liaocheng, China; b State Key Laboratory of Veterinary Biotechnology, Harbin Veterinary Research Institute, Chinese Academy of Agricultural Sciences, Harbin, China; c National Forestry and Grassland Administration, General Station for Surveillance of Wildlife Disease & Wildlife Borne Diseases, Shenyang, China; d Institute of Eco-Chongming (IEC), East China Normal University, Shanghai, China; Changchun Veterinary Research Institute

**Keywords:** H10N4, H10N8, avian influenza virus, wild birds, replication and transmission

## Abstract

H10Nx influenza viruses have caused increasing public concern due to their occasional infection of humans. However, the genesis and biological characteristics of H10 viruses in migratory wild birds are largely unknown. In this study, we conducted active surveillance to monitor circulation of avian influenza viruses in eastern China and isolated five H10N4 and two H10N8 viruses from migratory birds in 2020. Genetic analysis indicated that the hemagglutinin (HA) genes of the seven H10 viruses were clustered into the North American lineage and established as a novel Eurasian branch in wild birds in South Korea, Bangladesh, and China. The neuraminidase (NA) genes of the H10N4 and H10N8 viruses originated from the circulating HxN4 and H5N8 viruses in migratory birds in Eurasia. We further revealed that some of the novel H10N4 and H10N8 viruses acquired the ability to bind human-like receptors. Animal studies indicated that these H10 viruses can replicate in mice, chickens, and ducks. Importantly, we found that the H10N4 and H10N8 viruses can transmit efficiently among chickens and ducks but induce lower HA inhibition (HI) antibody titers in ducks. These findings emphasized that annual surveillance in migratory waterfowl should be strengthened to monitor the introduction of wild-bird H10N4 and H10N8 reassortants into poultry.

**IMPORTANCE** The emerging avian influenza reassortants and mutants in birds pose an increasing threat to poultry and public health. H10 avian influenza viruses are widely prevalent in wild birds, poultry, seals, and minks and pose an increasing threat to human health. The occasional human infections with H10N8 and H10N3 viruses in China have significantly increased public concern about the potential pandemic risk posed by H10 viruses. In this study, we found that the North American H10 viruses have been successfully introduced to Asia by migratory birds and further reassorted with other subtypes to generate novel H10N4 and H10N8 viruses in eastern China. These emerging H10 reassortants have a high potential to threaten the poultry industry and human health due to their efficient replication and transmission in chickens, ducks, and mice.

## INTRODUCTION

Influenza A viruses can be classified into subtypes based on the antigenicity and genetic properties of their membrane glycoproteins hemagglutinin (HA) and neuraminidase (NA). Currently, 16 HA subtypes and 9 NA subtypes of avian influenza viruses (AIVs) have been identified in wild birds and poultry. Wild birds, especially migratory waterfowl, are known as natural reservoirs of AIVs (1). AIVs in different wild birds pose an increasing threat to poultry and public health due to their worldwide prevalence and occasional cross-species infection (2). In the past 2 decades, the avian-origin H5N1, H5N6, H7N9, and H9N2 viruses have acquired the ability to infect mammals without prior adaptation and have led to more than 2,000 human infections in total (3–8). The H5Nx (H5N1, H5N2, H5N3, H5N5, H5N6, H5N8) viruses have evolved to form 10 clades and more than 20 subclades and subbranches and have continually reassorted with other subtypes of AIVs to generate different novel subtypes and genotypes (9–16). The worldwide spread of H5N8 viruses started in 2014, and the viruses have continually circulated in migratory wild birds in Europe, Asia, and Africa in recent years (9, 10, 17–20). Surveillance and monitoring of the prevalence of H5N8 viruses and their reassortment with other subtypes are urgently needed for detecting the emergence of novel reassortants.

H10Nx viruses, including the H10N3, H10N4, H10N7, and H10N8 viruses, have circulated in birds and mammals (minks and seals) in recent decades (21–25). H10 viruses have been reported to infect multiple avian species, and more than 1,600 H10 isolates have been identified and isolated from a wide range of wild birds (e.g., gulls, Anseriformes, and shorebirds), domestic waterfowl, and chickens (26–32). Some H10 isolates have adapted to infect mammals, as more than 60 H10N4 and H10N7 viruses have been isolated from minks and seals since 1984 (26, 33–35). An H10N5 virus was isolated from pigs in Hubei Province of central China in 2008 (36). More importantly, sporadic human infections with H10 viruses have caused increasing public concern because we are unlikely to have any prior immunity against these viruses (37). Three cases of human infection with H10N8 virus were first reported in China in 2013, of which two were fatal cases (38–40). In 2021, an avian-origin H10N3 virus caused human infection in China, resulting in severe pneumonia (41, 42). Liu et al. found that H10N3 reassortants isolated from chickens in eastern China showed high pathogenicity in mice and could be transmitted between chickens and guinea pigs (43). Fortunately, to date, there is no direct evidence of efficient human-to-human transmission of H10Nx viruses.

The HA genes of H10 viruses can be divided into North American and Eurasian lineages according to the phylogenetic tree of HA and their geographic prevalence (32). The prevalence of H10 viruses is closely related to the migratory flyway of wild birds. Generally, the H10 viruses of the North American lineage circulate predominantly in migratory birds in North America. However, the long migratory distance of some birds, such as gulls and Anseriformes, allows the viruses to be transmitted across the Asia-North America interface (19, 44–46). Additionally, the low pathogenicity of H10 viruses in migratory waterfowl does not usually cause explicit symptoms, allows them to replicate and persist in birds, and further increases the potential for the virus to develop mutations and undergo complex reassortment. Therefore, active monitoring of the intercontinental transmission of H10Nx viruses and identification of the reassortants of H10Nx viruses with other subtypes will contribute to early warnings for the invasion and spread of novel AIVs.

In this study, we conducted active surveillance in migratory birds in eastern China in 2020 to monitor the prevalence of different subtypes of AIVs and isolated five H10N4 and two H10N8 viruses. We performed a detailed analysis of their genetic evolution and phylogenetic relationships to reveal the emergence of novel H10N4 and H10N8 viruses. Biological studies on the novel H10N4 and H10N8 viruses revealed their replication, virulence, and transmission in birds and mammals.

## RESULTS

### Prevalence of H10N4 and H10N8 viruses in wild birds in eastern China in 2020.

We conducted active surveillance of AIVs in wetlands of the Yellow River Delta and wild swan habitats in eastern China in 2020 (Table 1). All the sampling sites are located in the East Asia-Australasia migratory flyway. We isolated 23 AIVs, including H1N1 (1), H2 (1), H3 (6), H4 (1), H5N8 (1), H7N7 (5), H9N2 (1), H10N4 (5), and H10N8 (2), from 1,820 samples of black-tailed gulls (Larus crassirostris), wild swans, and Eurasian coots (Fulica atra) and sequenced the whole genomes of the seven H10 viruses (the GenBank accession numbers are OM373211 to OM373226 and OM373267 to OM373306) (Table 1). Importantly, four H10N4 viruses and one H10N8 virus were detected in samples from the same wild swan population in the wetland of Swan Lake, suggesting that these two subtypes of influenza virus are simultaneously prevalent in wild swans (Table 1). Additionally, we isolated H10N4 and H10N8 viruses from gulls and Eurasian coots in the wetlands of the Yellow River Delta, which is another winter habitat of migratory birds in Shandong Province of eastern China. Notably, Eurasian coots are common breeding birds in lakes and streams in northern China and are widely distributed in the Palearctic, Middle East, and Indian subcontinent. Their global distribution and large population increase the chances of direct contact with domestic waterfowl. Accordingly, cocirculation of different subtypes of AIVs in migratory wild birds may prompt the cross-transmission of AIVs and emergence of novel reassortant viruses.

**TABLE 1:** Information of H10N4 and H10N8 avian influenza viruses isolated in this study

Virus no.	Full name	Abbreviation	Subtype	Isolation locations	Collection date	Host	Sample no.	Identified subtypes
1	A/black-tailed gull/Shandong/W1496/2020 (H10N8)	BTG/W1496/20	H10N8	Waterfowl habitats, Yellow River Delta, Shandong, China	15 March 2020	Black-tailed gull	314	H2(1), H3(1), H7N7(5), H10N8(1)
2	A/swan/Shandong/W3875/2020 (H10N8)	SW/W3875/20	H10N8	Wetland of Swan Lake, Shandong, China	23 November 2020	Wild swan	612	H1N1(1), H3N1(1), H3N8(2), H4(1), H5N8(1), H10N4(4), H10N8(1)
3	A/swan/Shandong/W3917/2020 (H10N4)	SW/W3917/20	H10N4					
4	A/swan/Shandong/W4047/2020 (H10N4)	SW/W4047/20	H10N4					
5	A/swan/Shandong/W4074/2020 (H10N4)	SW/W4074/20	H10N4					
6	A/swan/Shandong/W4322/2020 (H10N4)	SW/W4322/20	H10N4					
7	A/Eurasian coot/Shandong/W4446/2020 (H10N4)	EC/W4446/20	H10N4	Waterfowl habitats, Yellow River Delta, Shandong, China	29 November 2020	Eurasian coot	894	H3(2), H9N2(1), H10N4(1)

### Emergence of novel H10N4 and H10N8 reassortants.

**FIG 1:**
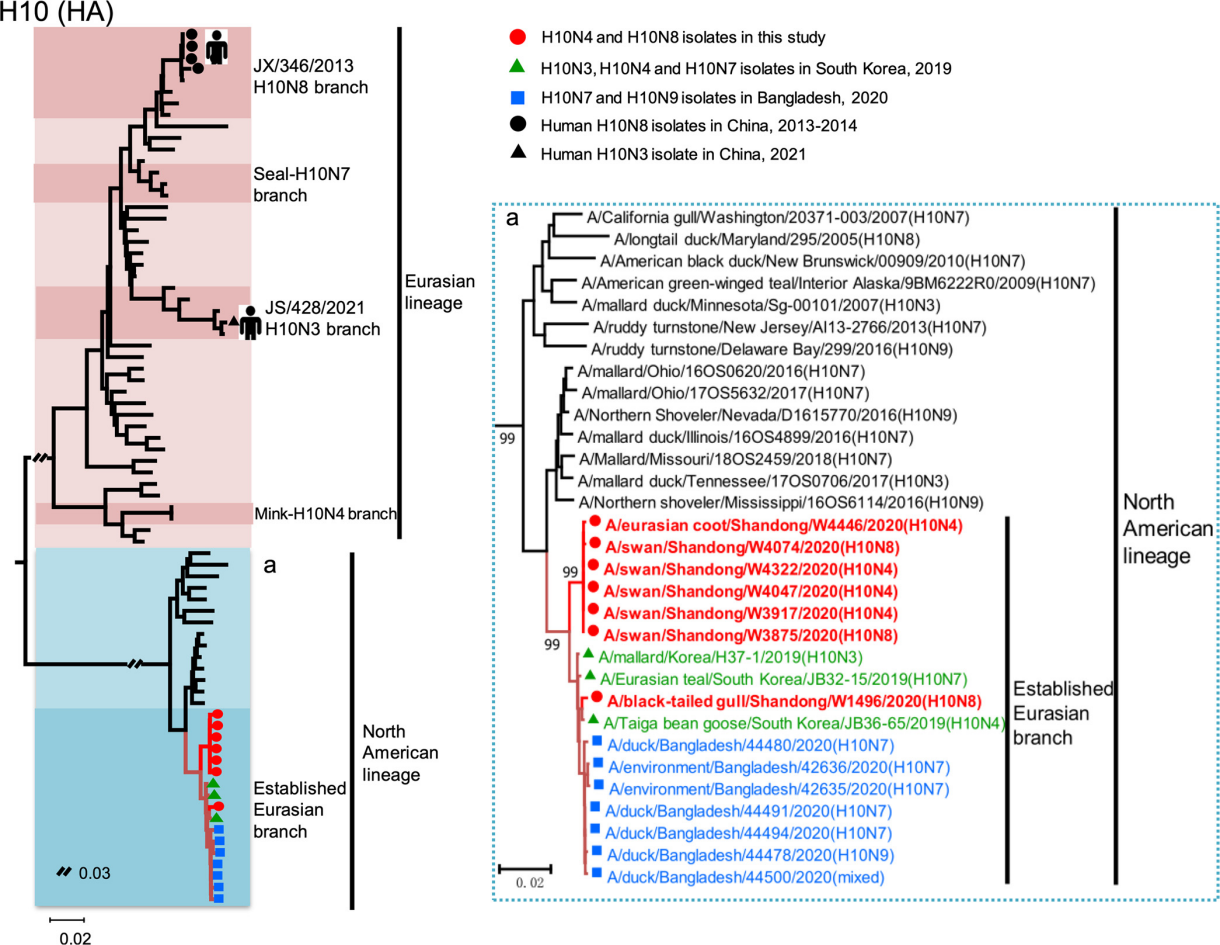
Phylogenetic tree of the HA genes of the H10 viruses. The phylogenetic tree was constructed by MEGA 7.0 with the neighbor-joining method. The sequence names in red are the viruses isolated in this study, and the sequences in green, blue, and black were downloaded from the database.

To clarify the genesis of the H10N4 and H10N8 viruses isolated from wild birds in this study, we first performed an evolutionary analysis of the HA and NA surface genes. We constructed a phylogenetic tree of HA genes of the seven H10 viruses with the reference sequences downloaded from database and found that the HA genes of the seven H10 viruses belonged to the Eurasian lineage. The HA genes of the viruses that have caused human infection, with the H10N3 virus in Jiangsu Province, China in 2021 and H10N8 viruses in Jiangxi Province, China in 2013 to 2014, formed two subbranches and clustered into the Eurasian lineage (Fig. 1). Interestingly, the HA genes of the seven H10 isolates in this study clustered in the North American lineage and established a novel Eurasian branch with the H10N3, H10N4, H10N7, and H10N9 viruses that circulated in migratory wild birds and ducks in South Korea and Bangladesh in 2019 and 2020 (Fig. 1). The phylogenetic tree of the HA gene and the BLAST results indicated that H10 viruses of the newly established Eurasian branch of the North American lineage first emerged in South Korea in 2019 and then spread to other Asian countries, including Bangladesh and China (Fig. 1).

**FIG 2:**
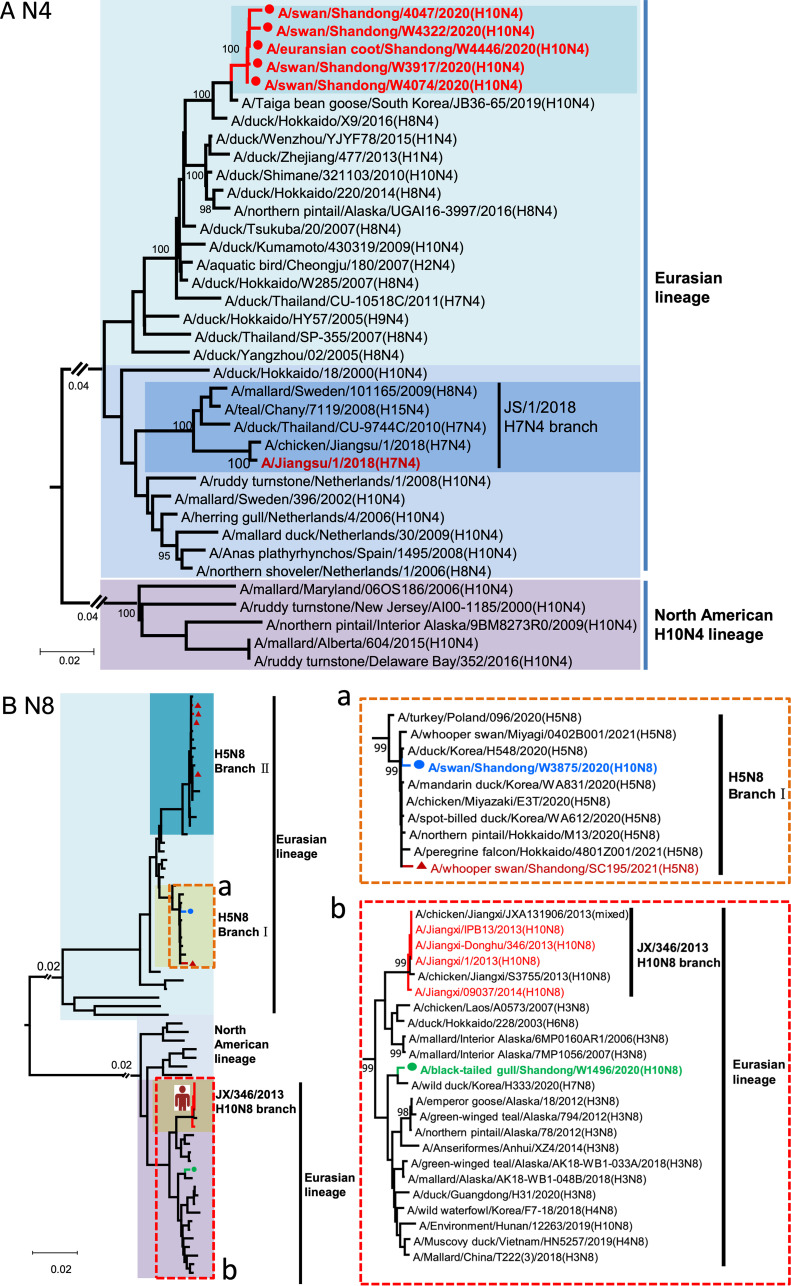
Phylogenetic analysis of the NA genes of the H10N4 and H10N8 viruses. (A) NA phylogenetic tree of H10N4 viruses. (B) NA phylogenetic tree of H10N8 viruses. The sequence names in green and blue indicate the H10N8 viruses we isolated in this study, and the sequence of A/whooper swan/Shandong/SC195/2021 (H5N8) is that of the H5N8 virus isolated in our previous study. The phylogenetic tree was constructed by MEGA 7.0 with the neighbor-joining method.

The Eurasian lineage NA genes of the five H10N4 viruses shared 99.2 to 99.9% nucleotide identity and were closest to the taiga bean goose isolate in South Korea in 2019 (Fig. 2A). The NA gene of BTG/W1496/20 H10N8 was closest to the H3N8, H4N8, and H10N8 viruses that circulated in wild birds and clustered into the Eurasian N8 NA lineage with the JX/346/2013-like H10N8 viruses. The NA gene of the SW/W3875/20 H10N8 was closest to the circulating H5N8 viruses, and they clustered in H5N8 branch I with the clade 2.3.4.4b H5N8 viruses detected in wild birds in East Asia in 2020 and 2021 (Fig. 2B). Phylogenetic analysis indicated that the six internal genes of the H10N4 and H10N8 viruses originated from the wild-bird viruses that circulated in the Eurasian region. In particular, the wild-bird H9N2-like viruses that circulated in Shandong Province in 2019 contributed to the emergence of the reassortant H10N4 and H10N8 viruses as gene donors (Fig. S1 and S2) (4). Accordingly, SW/W3875/20 is a novel H10N8 reassortant of H10Nx, H5N8, H9N2-like, and other wild-bird viruses. BTG/W1496/20 is a reassortant of North American lineage H10Nx viruses, Eurasian lineage HxN8 viruses, H9N2-like viruses, and other wild-bird viruses. The five H10N4 viruses and the South Korean H10N4 viruses are reassortants of North American lineage H10Nx viruses, Eurasian lineage HxN4 viruses, H9N2-like viruses, and other wild-bird viruses (Fig. 2, Fig. S1 and S2). According to the phylogenetic analysis of each gene segment of the seven viruses, we then divided the two H10N8 viruses into two genotypes and the five H10N4 viruses into one genotype (the distance between group in each phylogenetic tree was lower than 95%) (Fig. S2A).

**FIG 3:**
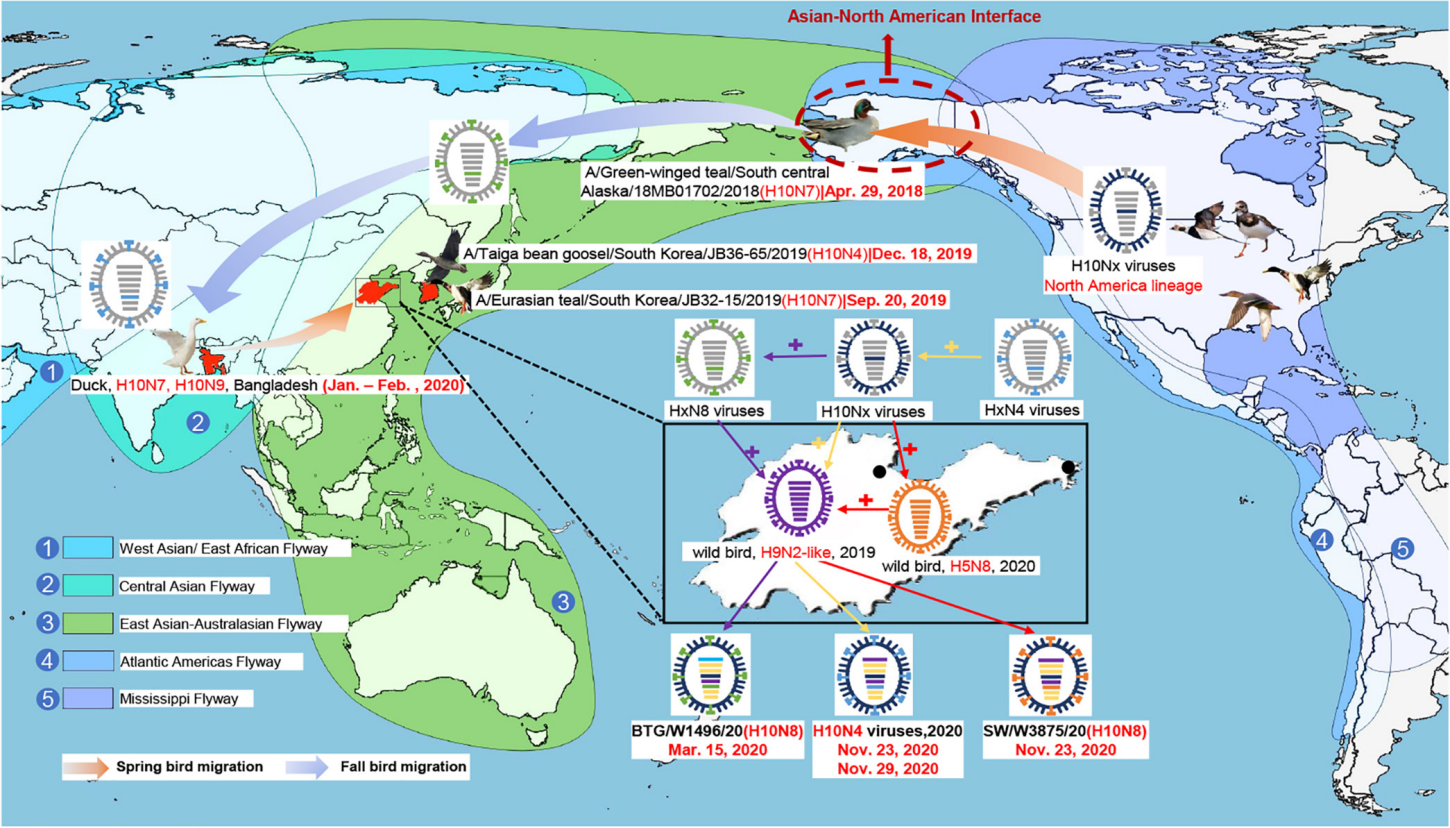
Proposed transmission and reassortment of H10Nx viruses in wild migratory birds in North America and Eurasia. The transmission routes of the HA gene of the H10Nx viruses are indicated with arrows. Sampling points are marked with black circles. The migratory flyways are marked with different colors. Wild-bird H9N2-like viruses were isolated in the Yellow River Delta in eastern China in 2019.

Based on these findings, we proposed a possible evolutionary pathway for the H10 HA gene of the newly established Eurasian sublineage. The H10Nx viruses of the North American lineage were first transmitted to Alaska (overlapping breeding regions of the East Asia-Australasia migratory flyway and Atlantic Americas flyway) by migratory waterfowl in 2018 and then migrated to South Korea with migratory birds in the fall of 2019, followed by detection in overwintering migratory birds in Bangladesh in 2020. The viruses were then transmitted to eastern China during the spring migration of migratory birds, and finally, the H10N4 and H10N8 viruses were detected in wintering migratory birds in this study in 2020 (Fig. 3).

### Receptor-binding specificity and antigenic analysis of novel H10N4 and H10N8 isolates.

**FIG 4:**
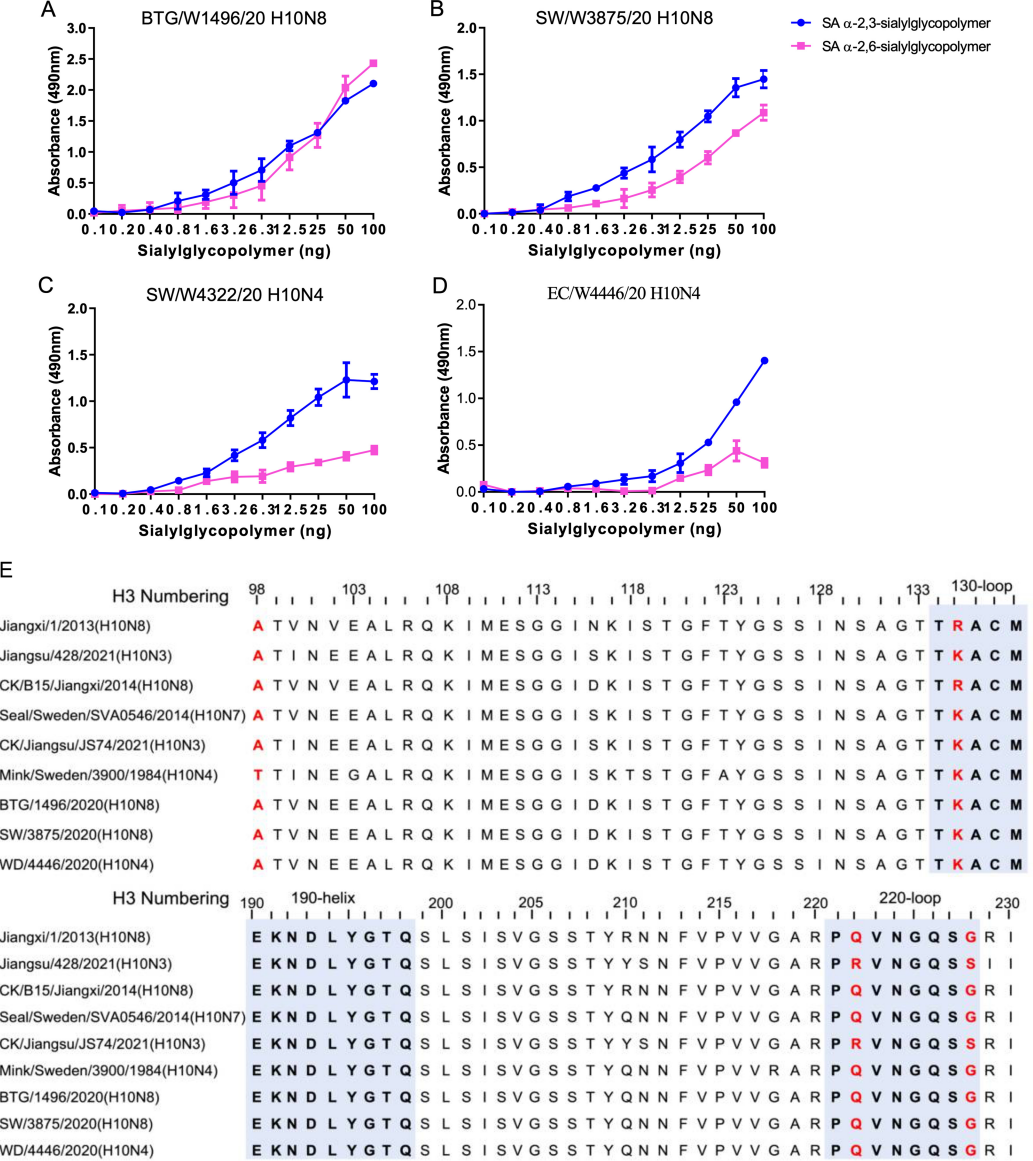
Receptor-binding properties and sequence differences in the receptor-binding domain of HA of H10N4 and H10N8 viruses. (A to D) Receptor-binding specificity of H10N4 and H10N8 viruses. Two different glycopolymers (α-2,3-siaylglycopolymer and α-2,6-siaylglycopolymer) were used to test the receptor-binding properties of the viruses. The data shown are the means of three replicates; the error bars indicate the standard deviation. (E) Comparison of the amino acids of the receptor-binding region in HA of the representative wild bird, chicken, mink, seal, and human H10 isolates.

Based on the genetic analysis of the wild bird-origin H10N4 and H10N8 reassortants, we performed further evaluation of the receptor-binding specificity of these novel H10 viruses. Here, we used α-2,3-sialylglycopolymer (an avian-like receptor) and α-2,6-sialylglycopolymer (a human-like receptor) to characterize the receptor binding of the novel H10N4 and H10N8 viruses in this study. Interestingly, the BTG/W1496/20 and SW/W3875/20 H10N8 viruses bound to both the avian-like receptor and human-like receptor, while the SW/W4322/20 and EC/W4446/20 H10N4 viruses preferentially bound to the avian-like receptor (Fig. 4A to E). We then aligned the HA sequences to compare their amino acid sequences with those of human, mink, seal, and chicken H10 isolates and found that the amino acids 135K, 222Q, and 228G in the receptor-binding region were different from those in the human H10N3 or H10N8 isolates (Fig. 4E, Table S1). To evaluate the antigenic differences in the H10N4 and H10N8 viruses used in this study, we performed an HA inhibition (HI) assay by using antisera generated in specific pathogen-free (SPF) chickens. The HI titers against the homologous viruses and different H10N4 or H10N8 strains ranged from 32 to 256, which suggested that the H10N4 and H10N8 viruses shared similar antigenicity (Table S2). These results indicated that the novel H10N4 and H10N8 reassortants preferentially bind to avian-like receptors and that some have acquired the ability to bind human receptors.

### Novel H10N4 and H10N8 reassortants can replicate in both chicken and mammalian cells.

We then evaluated the replication of the H10N4 and H10N8 viruses in avian and mammalian cells. The tested virus was inoculated into the cells in 24-well plates, and the culture supernatants of chicken embryo fibroblasts (CEFs), MDCK cells, and human A549 cells were collected and titrated in eggs. Interestingly, a significant difference in the replication of H10N4 and H10N8 viruses in avian and mammalian cells was observed. The titers of the four tested viruses in MDCK cells were significantly higher than those in CEFs and A549 cells (Fig. S3). These results suggested that the wild bird-originated H10N4 and H10N8 viruses replicated poorly in chicken and human cells.

### Replication and virulence of H10N4 and H10N8 viruses in mice.

**FIG 5:**
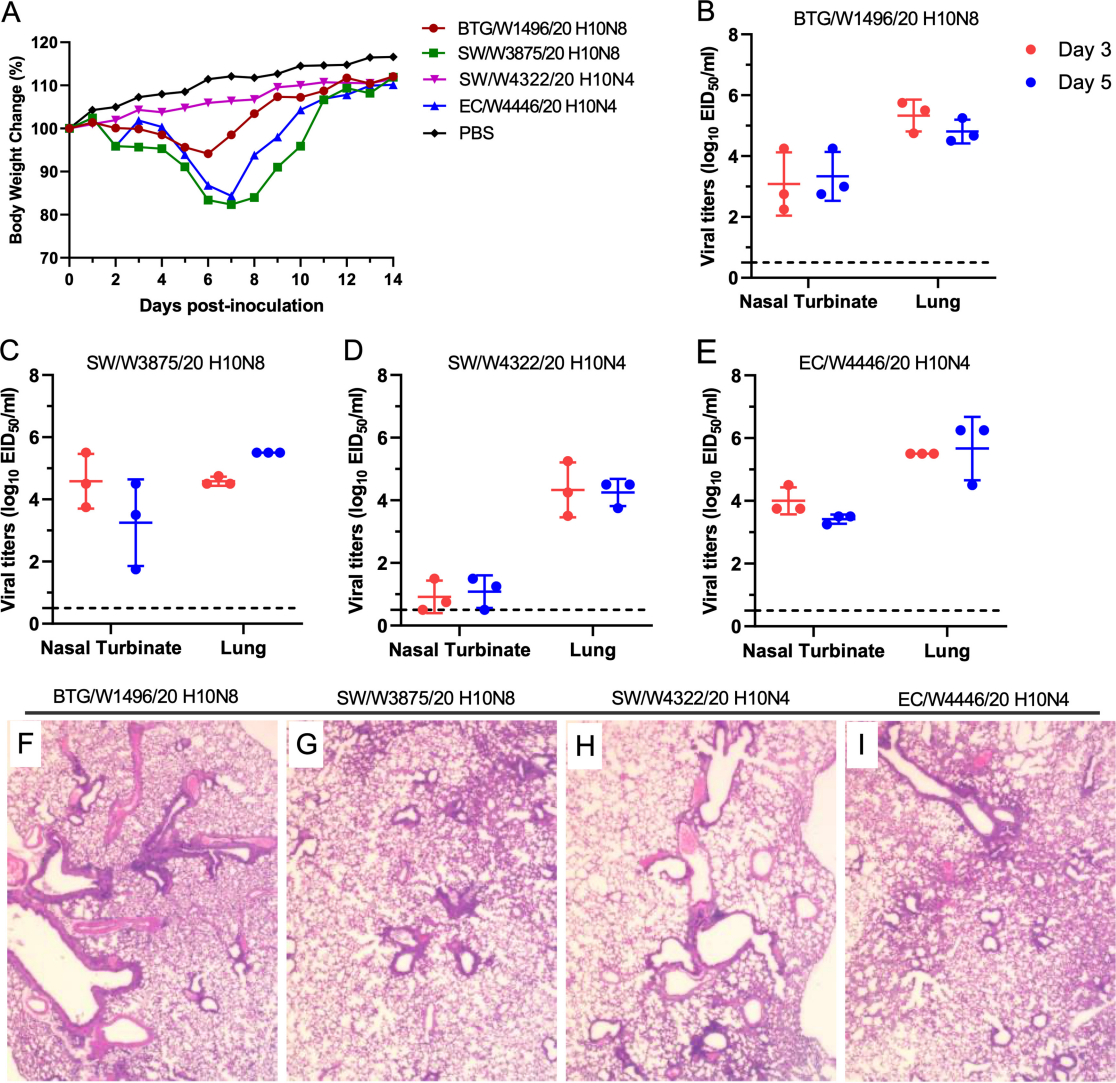
Replication, virulence, and pathological lesions of H10N4 and H10N8 isolates in mice. (A) Body weight change of the mice infected with H10N4 and H10N8 isolates. (B to E) Viral titers in mice inoculated with 10^6^ EID_50_ of the viruses. (A) BTG/W1496/20 H10N8, (B) SW/W3875/20 H10N8, (C) SW/W4322/20 H10N4, (D) EC/W4446/20 H10N4. (F to I) Pathological lesions of the lungs of mice inoculated with the viruses. (F) BTG/W1496/20 H10N8, (G) SW/W3875/20 H10N8, (H) SW/W4322/20 H10N4, (I) EC/W4446/20 H10N4.

To date, H10N3 and H10N8 viruses have been reported to infect humans, and several studies have also revealed that poultry-originated H10N3 and H10N8 viruses can replicate efficiently in mice (43, 47). To understand the virulence of wild bird-originated H10N4 and H10N8 reassortants, we tested four representative isolates in mice. We found that three viruses, namely, EC/W4446/20 H10N4, BTG/W1496/20 H10N8, and SW/W3875/20 H10N8, replicated efficiently in the nasal turbinates and lungs of mice and caused body weight loss from 5.9% to 17.6% in the mice. However, SW/W4322/20 H10N4 replicated poorly in the nasal turbinates of mice and did not cause body weight loss (Fig. 5A to E). The pathological analysis indicated that the H10N4 and H10N8 viruses caused mild to moderate lung lesions (Fig. 5F to I). These results indicate that the novel H10N4 and H10N8 viruses isolated from migratory wild birds can replicate in mice and lead to significant clinical symptoms without prior adaptation.

### Replication of H10N4 and H10N8 viruses in chickens and ducks.

**FIG 6:**
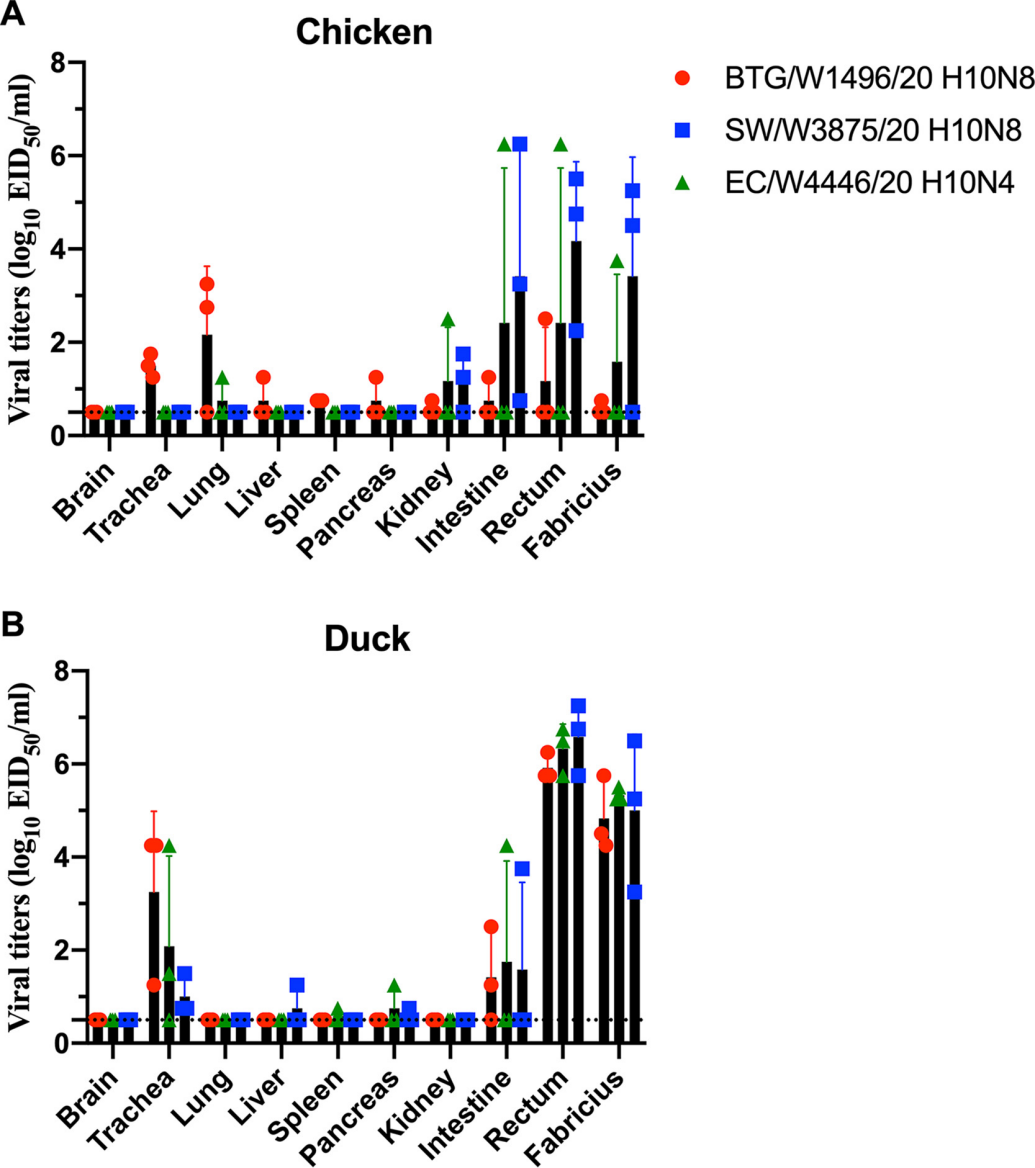
Replication of H10N4 and H10N8 isolates in chickens and ducks. Viral titers of BTG/W1496/20 H10N8, SW/W3875/20 H10N8, and EC/W4446/20 H10N4 in the organs of chickens (A) and ducks (B) at day 3 p.i. inoculated with 10^6^ EID_50_ of the test virus. The data shown are the mean with SD of the titers from three chickens or ducks. The dashed line indicates the lower limit of detection.

The replication of the novel H10N4 and H10N8 viruses in chickens and ducks is poorly understood. To investigate the replication of the H10N4 and H10N8 viruses in poultry, we tested the BTG/W1496/20, SW/W3875/20, and EC/W4446/20 viruses in chickens and ducks. After intranasal inoculation with the tested viruses with 10^6^ 50% egg infective dose (EID_50_), three chickens were euthanized at 3 days postinfection (dpi) to detect viral titers in different organs. As shown in Fig. 6A, all three viruses replicated efficiently in chickens, with viral titers in different organs ranging from 10^0.75^ EID_50_ mL^−1^ to 10^6.25^ EID_50_ mL^−1^. The BTG/W1496/20 H10N8 virus preferentially replicated in the lung and trachea of chickens, with limited replication titers in other tested organs. However, the EC/W4446/20 H10N4 and SW/W3875/20 H10N8 viruses replicated primarily in the intestinal canal and bursa of Fabricius of the infected chickens, with lower viral titers in the lung and trachea. The viruses could be detected in kidneys with lower viral titers in chickens. No virus or very limited virus was detected in the brain, liver, spleen, or pancreas of the infected chickens (Fig. 6A). The organs of the inoculated ducks were collected at 3 dpi to test viral replication in eggs. The three H10 viruses could be detected in the trachea but not in the lungs of inoculated ducks, indicating that these H10 viruses need prior adaptation to replicate efficiently in the lower respiratory tract of ducks. Interestingly, the viruses were detected in the rectum and bursa of Fabricius in inoculated ducks with high viral titers. The viruses could be detected in the intestine in all groups of ducks with relatively low viral titers. No virus or very limited virus was detected in the brain, liver, spleen, pancreas, or kidney of the infected ducks (Fig. 6B). In summary, we showed that the wild-bird H10N4 and H10N8 viruses can replicate in both chickens and ducks.

### Transmission of H10N4 and H10N8 viruses in chickens and ducks.

**FIG 7:**
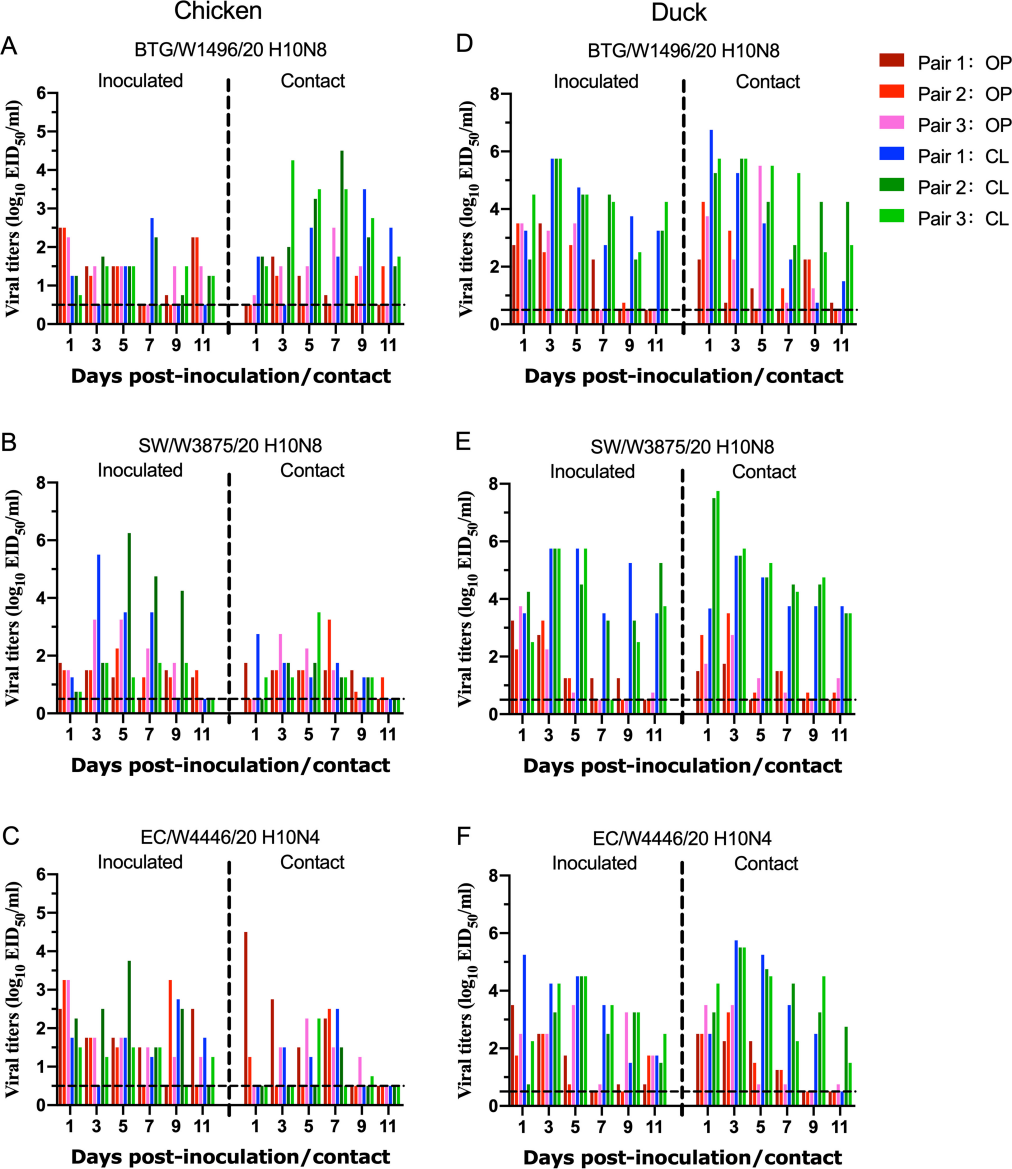
Transmission of H10N4 and H10N8 viruses in chickens and ducks. Groups of SPF chickens and ducks (*n* = 3) were inoculated intranasally with 10^6^ EID^50^ virus in a volume of 200 μL. Three naive chickens or ducks of the contacted group were housed in the same isolator as the contact birds at 24 hpi. The oropharyngeal and cloacal swabs of the animals were suspended in 1 mL of PBS and titrated for viral shedding in eggs. (A to C) Transmission study of H10N4 and H10N8 viruses in chickens. (D to F) Transmission study of H10N4 and H10N8 viruses in ducks. The dashed line indicates the lower detection limit.

After we uncovered the replication of the novel H10N4 and H10N8 viruses in chickens and ducks, we further tested their transmission ability in both chickens and ducks. Oropharyngeal and cloacal swabs of the chickens in the infected or contacted groups were collected at 1, 3, 5, 7, 9, and 11 dpi and titrated in eggs. As shown in Fig. 7A to C, all three tested viruses were detected in the swabs of inoculated and contacted chickens. In the BTG/W1496/20 H10N8 and SW/W3875/20 H10N8 virus test groups, the virus was detected in both inoculated and contacted chickens from 1 to 11 dpi, with titers ranging from 10^0.75^ to 10^4.25^ EID_50_ mL^−1^ and 10^0.75^ to 10^6.25^ EID_50_ mL^−1^, respectively. In the EC/W4446/20 H10N4 virus test group, the virus was detected in swabs in the inoculated chickens from 1 to 11 dpi, and the virus could be transmitted to three naive chickens with viral titers ranging from 10^0.75^ to 10^4.5^ EID_50_ mL^−1^ in the contacted group from 1 to 9 dpi (Fig. 7A to C). The three viruses could be detected in oropharyngeal and cloacal swabs of both inoculated and contacted ducks from 1 to 11 days postinfection (dpi). In the BTG/W1496/20 H10N8 and SW/W3875/20 H10N8 virus test groups, the viral titers in cloacal swabs of the contact ducks were up to 10^6.75^ and 10^7.75^ EID_50_ mL^−1^, respectively. The relatively high viral titers and persistent viral shedding period in cloacal swabs indicated that the H10 viruses could be transmitted efficiently to naive ducks by cloacal virus shedding (Fig. 7D to F). Importantly, the viral titers of oropharyngeal and cloacal swabs of the inoculated and contacted ducks were significantly higher than those of the chickens, which indicated that the wild-bird H10 viruses preferentially replicated in ducks rather than in chickens.

We then detected the HI antibody in chicken and duck antisera to reveal the immune response against H10 virus infection. Chicken and duck sera were collected at 10, 15, and 21 dpi, and the HI antibody in each serum was detected by cross-reaction with the three H10 viruses. The HI antibody titers of the chickens infected with the H10 viruses ranged from 16 to 256; however, the HI antibody titers of the two contacted chickens in the EC/W4446/20 H10N4 virus test group were negative, although the virus could be detected in the contacted chickens (Table S3). Unlike the HI antibody dynamics of the chickens, the HI antibody titers of the ducks were relatively low. The HI antibody titers of the ducks infected with the H10 viruses ranged from 8 to 128. Importantly, the HI antibody titers of the ducks at 21 dpi were lower than those at 10 and 15 dpi (Table S3). In summary, these results indicate that the H10N4 and H10N8 viruses used in this study could be transmitted efficiently in both chickens and ducks, and infection with the H10 viruses could not induce the production of high HI antibody titers in ducks.

## DISCUSSION

In this study, we performed active surveillance to monitor the cocirculation of different subtypes of AIVs in wild birds in eastern China. The H10N4 and H10N8 viruses were detected in gulls, wild swans, and Eurasian coots in wetlands located in the East Asian-Australasian migratory flyway. Genetic analysis indicated that North American lineage H10Nx viruses have been successfully introduced into the Eurasian region by migratory birds. More importantly, these introduced H10Nx viruses further reassorted with the prevalent H5N8 viruses and HxN4 viruses to generate the novel H10N4 and H10N8 viruses. These viruses have successfully established a new Eurasian branch with H10N3, H10N4, H10N7, and H10N9 viruses detected in wild birds and ducks in South Korea and Bangladesh in 2019 and 2020. Some of these novel H10 viruses could bind both avian- and human-like receptors and replicate efficiently in mice, leading to significant body weight loss in infected mice. Notably, the wild bird-originated H10N4 and H10N8 viruses replicated efficiently and could transmit in chickens and ducks.

In the past 2 decades, emerging and reemerging animal influenza viruses and other zoonoses have posed major challenges to public health worldwide (48–51). Widely circulated wild-bird AIVs can be introduced into domestic poultry by migratory birds and domestic waterfowl, thereby increasing the risk of cross-species transmission between birds and mammals. The H5N8 viruses continually circulate in migratory birds in Asia, Europe, North America, and Africa and are highly likely to generate novel mutants by reassortment with other subtypes (52–54). Currently, the H10 viruses are divided into Eurasian and North American lineages and are widely circulating in both wild migratory birds and domestic poultry (32, 46). We found here that North American lineage H10 viruses have been introduced into migratory birds in Asia and generated novel H10N4 and H10N8 viruses by reassorting with current HxN4 and H5N8 viruses in migratory birds. The ongoing surveillance in wild-bird habitats, live poultry markets, and farms will contribute to monitoring the introduction and circulation of these H10N4 and H10N8 viruses.

H10 viruses are a growing animal health and public health concern, not only because the viruses are widely circulating in birds and mammals but also because they can infect humans at the avian-human interface. A previous study found that the avian-origin H10N8 viruses that infected humans in 2013 in China retained a strong preference for avian-type receptors (55), but another study reported that the H10 virus possessed high avidity for human-type receptors (56). Here, we found that wild-bird H10N4 and H10N8 viruses have acquired the ability to bind the human-like receptor and caused significant body weight loss in infected mice, which implied that circulating H10N4 and H10N8 reassortants can replicate in mice without prior adaptation. The receptor-binding preference of the H10N4 and H10N8 viruses used in this study was consistent with that of the previously reported chicken H10N3 viruses, which showed high pathogenicity and respiratory droplet transmissibility in mammals (43). Therefore, continuous studies are essential to monitor the mutations of the receptor-binding specificity and the enhanced virulence of the emerged H10N4 and H10N8 viruses in mammals.

Herein, we tested the replication and transmission of the H10N4 and H10N8 viruses and found that wild-bird H10 viruses can replicate and be transmitted efficiently in chickens and ducks. In particular, H10 viruses are preferentially replicated in ducks over chickens, which suggests that domestic ducks are susceptible hosts of H10 viruses. However, duck studies indicate that H10 virus infection cannot induce the production of high titers of the HI antibody in ducks. The titers of the HI antibody in chickens were higher than those in ducks, but the sera of two chickens in the EC/W4446/20 group gave a negative result, although we detected viral replication in the chickens. Additionally, the H10 isolates used in this study preferentially replicated in the enteric canal and bursa of Fabricius of the chickens and ducks. These replication and transmission studies in chickens and ducks indicated that circulating novel H10 viruses in migratory birds have a high potential risk of transmission to domestic poultry introduced by fecal droppings or the contaminated environment. The silent replication of H10 viruses in birds without clinical symptoms contributes to the generation of novel reassortant progeny viruses with other predominant subtypes, such as the widely prevalent H9N2 viruses in wild birds and poultry (4, 57). In summary, active surveillance in both wild birds and poultry will contribute to the monitoring of the invasion and prevalence of the H10N4 and H10N8 viruses in migratory waterfowl and poultry, especially for reducing the potential transmission risk of these H10 reassortants from birds to humans.

## MATERIALS AND METHODS

### Ethics statements and facility.

The animal studies were carried out in strict accordance with the recommendations in the Guide for the Care and Use of Laboratory Animals of the Ministry of Science and Technology of the People’s Republic of China. The protocols for chicken, duck, and mouse studies were approved by the Committee on the Ethics of Animal Experiments of the College of Agronomy of Liaocheng University. All experiments with live H10N4 and H10N8 viruses were conducted within the animal biosafety level 2 (ABSL-2) facility at Liaocheng University. The animals used in this study were placed in a biological safety isolator. The researchers who worked with the mice, chickens, and ducks wore N95 masks and disposable overalls.

### Eggs and cells.

SPF embryonated chicken eggs were obtained from Jinan SPFR Poultry Industry Science and Technology, Ltd. Madin–Darby canine kidney (MDCK) and A549 cells were purchased from the Cell Resource Center of the Shanghai Institute of Life Sciences. Chicken embryo fibroblasts (CEFs) were obtained from 10-day-old SPF embryonated chicken eggs. MDCK cells and CEFs were grown in minimum essential medium with Eagle salts containing 10% fetal bovine serum, 4 mM l-glutamine, and antibiotics. The A549 cells were maintained in F-12K nutrient mixture supplemented with 10% fetal bovine serum plus antibiotics. All cells were incubated at 37°C in 5% CO_2_.

### Experimental animals.

Six-week-old SPF female BALB/c mice were purchased from Jinan Pengyue Experimental Animal Breeding Co., Ltd., Shandong, China. Six-week-old SPF chickens and 3-week-old ducks were purchased from Shandong Healthtech Laboratory Animal Breeding Co., Ltd.

### Sampling, virus identification, and isolation.

Fresh droppings of wild swans, Eurasian coots (Fulica atra), wild ducks, and other wild waterfowl in the habitats were collected and then placed into 2 mL of minimal essential medium supplemented with penicillin and streptomycin. The samples were first detected by PCR with specific M and HA (H5, H7) primers. The suspected H5- or H7-positive samples were transferred to the enhanced animal biosafety level 3 facility in the HVRI of the CAAS for further diagnosis and virus isolation, while the remaining suspected positive samples were injected into 10-day-old embryonated chicken eggs to isolate the viruses in the animal biosafety level 2 (ABSL-2) laboratory at Liaocheng University. The HA subtype was determined by using the HA inhibition (HI) test, and the neuraminidase (NA) subtype was determined by PCR and genetic sequencing. The viruses were stored in a −80°C freezer, and the virus stocks were grown in SPF chicken eggs.

### Molecular and phylogenetic analyses.

The RNA of the viruses was extracted from the allantoic fluid of virus-infected eggs, and then, reverse transcription-PCR was performed by using gene-specific primers. The PCR products of eight segments of H5N8, H10N4, and H10N8 viruses were sequenced by specific sequencing primers (primer sequences available on request). The sequence data were compiled with the SEQMAN program (DNASTAR, Madison, WI) according to the reference sequences. Phylogenetic analysis was performed by employing the maximum likelihood method using the MEGA 7.0 ClustalW software package. The phylogenetic tree was constructed with the PHYLIP program of MEGA 7.0 software using the neighbor-joining algorithm. A bootstrap value of 1,000 was used.

### Receptor-binding assay.

We used a solid-phase direct binding assay to examine the receptor-binding property of the viruses as described previously (4, 5). Here, two glycopolymers, α-2,3-siaylglycopolymer (Neu5Aca2-3Galb1-4GlcNAcb1-pAP [para-aminophenyl]-alpha-polyglutamic acid [α-PGA]) and α-2,6-sialylglycopolymer (Neu5Aca2-6Galb1-4GlcNAcb1-pAP [para-aminophenyl]-alpha-polyglutamic acid [α-PGA]), were used to test the receptor-binding specificity of the H10N4 and H10N8 viruses. A specific chicken anti-HA polyclonal antibody was used to react with the tested viruses. Dose-response curves of virus binding to the glycopolymers were analyzed by using a single-site binding algorithm and curve fitting by GraphPad Prism 8 to determine the associated constant values (Ka). Each value is presented as the mean ± standard deviation (SD) of three independent experiments, each of which was performed in triplicate.

### Curves for viral replication in cells.

The viruses were diluted to 10^6^ EID_50_/200 μL in OPTI-MEM containing 0.5 μg/mL trypsin-tosylsulfonyl phenylalanyl chloromethyl ketone (TPCK) and then inoculated in monolayer cells in 24-well plates. One hour postinfection (hpi), the infected cells were washed with phosphate-buffered saline (PBS) three times, and 500 μL of fresh OPTI-MEM containing 0.5 μg/mL trypsin-TPCK was added. The supernatant was collected in a 1.5 mL centrifuge tube at 12, 24, 48, and 72 hpi and titrated into eggs. The growth curves shown are the average results of three independent experiments.

### Chicken study.

Twenty-seven 6-week-old SPF chickens were divided into three groups to test the replication of the BTG/W1496/20 H10N8, SW/W3875/20 H10N8, and EC/W4446/20 H10N4 viruses in chickens. The chickens were inoculated with 10^6^ EID_50_ of the virus in a volume of 200 μL. The brain, trachea, lung, liver, spleen, pancreas, kidneys, intestine, rectum, and bursa of Fabricius were collected for viral titration in eggs at day 3 postinoculation.

For the transmission study, three chickens of the inoculated group were inoculated with 10^6^ EID_50_ of the virus in a volume of 200 μL. Another three naive chickens of the contact group were placed into the same isolator at 24 hpi. The oropharyngeal and cloacal swabs of the chickens were collected on days 1, 3, 5, 7, 9, and 11 postinfection (p.i.), respectively. The viral titers of the swabs were titrated in eggs. Chicken serum was collected on days 10, 15, and 21 p.i., and the antibody titer was determined by the HI test. The chickens were then euthanized on day 21 p.i.

### Duck study.

Twenty-seven 3-week-old SPF ducks were divided into three groups to test the replication of the BTG/W1496/20 H10N8, SW/W3875/20 H10N8, and EC/W4446/20 H10N4 viruses in ducks. The methods for virus inoculation, collection, and titration of the organs, collection of the oropharyngeal and cloacal swabs, collection of duck serum, and the HI test were the same as those used for the chicken study as described above.

### Mouse study.

Six-week-old female SPF mice (14 animals in each group) were inoculated with 10^6^ EID_50_ of the virus in a volume of 50 μL, three mice were euthanized on days 3 and 5 p.i., and the nasal turbinate, lung, spleen, kidney, and brain were collected for viral titration in eggs. Three mice were euthanized on day 3 p.i., and the lungs were fixed in 10% formalin and then stained with hematoxylin and eosin (H&E) for histological analysis. The remaining five mice were monitored daily for 14 days for weight loss and survival. Mice inoculated with PBS were established as a control group and used to observe body weight and pathological changes.

### Statistical analysis.

The data were statistically analyzed with one-way analysis of variance (ANOVA) followed by a *t* test by using GraphPad Prism 8.0 software. Statistical parameters are marked in the figures and figure legends. A *P* value of <0.05 was considered statistically significant.

### Data availability.

We isolated 23 AIVs, including H1N1 (1), H2 (1), H3 (6), H4 (1), H5N8 (1), H7N7 (5), H9N2 (1), H10N4 (5), and H10N8 (2), from 1,820 samples of black-tailed gulls (Larus crassirostris), wild swans, and Eurasian coots (Fulica atra). The sequences of the whole genomes of the seven H10 viruses have been submitted to GenBank (the GenBank accession numbers are OM373211 to OM373226 and OM373267 to OM373306).
