# Supplementary material for: Emergence, Evolution, and Biological Characteristics of H10N4 and H10N8 Avian Influenza Viruses in Migratory Wild Birds Detected in Eastern China in 2020
Source: Microbiol Spectr. 2022 Apr 7;10(2):e00807-22. doi: 10.1128/spectrum.00807-22 (PMC9045299; doi:10.1128/spectrum.00807-22)
Supplement: SUPPLEMENTAL FILE 1 — Supplemental material. Download SPECTRUM00807-22_Supp_1_seq11.pdf, PDF file, 2.5 MB [file spectrum00807-22_supp_1_seq11.pdf]

Table S1. Amino acids substitutions of the H10 viruses that contribute to the enhanced replication, pathogenicity, and transmissibility in mammals.

Viruses	HA (H3 Number)				PB2			PB1		PA		M1		NS1
	Q226L	G228S	N379T	Cleavage site	G309D	E627K	D701N	K328N	S375N	E382D	A515T	N30D	T215A	P42S
BTG/W1496/20	Q	G	T	VPEVVQG.GLF	D	E	D	N	N	D	T	D	A	A
SW/W3875/20	Q	G	T	VPEVVQG.GLF	D	E	D	N	N	D	T	D	A	S
SW/W3917/20	Q	G	T	VPEVVQG.GLF	D	E	D	N	N	D	T	D	A	S
SW/W4047/20	Q	G	T	VPEVVQG.GLF	G	E	D	N	N	D	T	D	A	S
SW/W4074/20	Q	G	T	VPEVVQG.GLF	D	E	D	N	N	D	T	D	A	S
SW/W4322/20	Q	G	T	VPEVVQG.GLF	D	E	D	N	N	D	T	D	A	S
EC/W4446/20	Q	G	T	VPEVVQG.GLF	D	E	D	N	N	E	T	D	A	S

Table S2. Cross-reactive hemagglutinin inhibition (HI) antibody titers of the H10viruses with different chicken antisera.

viruses	HI antibody titer of chicken antiserum [a]		
	BTG/1496/20	SW/3875/20	EC/4446/20
BTG/1496/20 H10N8	**128** [c]	64	32
SW/3875/20 H10N8	32	**256**	64
EC/4446/20 H10N4	32	128	**128**
SW/4322/20 H10N4	128	128	64

[a] The antisera were generated by infecting SPF chickens with the H10 viruses, as indicated in this table.

[c] The homologous titer is shown in bold.

Table S3. Seroconversion of the chickens and ducks inoculated or contacted with H10N4 and H10N8 influenza viruses

Animals	Viruses	Seroconversion: positive/total [a] (HI antibody titer)					
		Inoculated group (dpi [b])			Contact group (dpc[c])		
		10	15	21	10	15	21
Chicken	BTG/1496/20	3/3<br>(64, 64, 256)	3/3<br>(64, 64, 256)	3/3<br>(64, 128, 128)	3/3<br>(16, 64, 128)	3/3<br>(32, 64, 64)	3/3<br>(128, 128, 128)
	SW/3875/20	3/3<br>(128, 256, 256)	3/3<br>(128, 128, 256)	3/3<br>(128, 128, 256)	3/3<br>(16, 16, 64)	3/3<br>(32, 64, 64)	3/3<br>(64, 64, 128)
	EC/4446/20	3/3<br>(64, 128, 128)	3/3<br>(32, 32, 64)	3/3<br>(128, 128,128)	1/3<br>(- [d], -, 16)	1/3<br>(-, -, 64)	1/3<br>(-, -, 128)
Duck	BTG/1496/20	3/3<br>(16, 16, 32)	3/3<br>(16, 16, 16)	3/3<br>(8, 8, 8)	3/3<br>(8, 8, 16)	3/3<br>(16, 16, 32)	3/3<br>(4, 16, 16)
	SW/3875/20	3/3<br>(32, 32, 64)	3/3<br>(16, 32, 128)	3/3<br>(16, 32, 32)	3/3<br>(8, 16, 16)	3/3<br>(8, 32, 32)	2/3<br>(-, 2, 8)
	EC/4446/20	3/3<br>(32, 64, 64)	3/3<br>(32, 32, 64)	3/3<br>(16, 32, 32)	3/3<br>(8, 8, 16)	3/3<br>(8, 8, 16)	3/3<br>(2, 2, 4)

[a] The chicken or the duck serum was collected on days 10, 15, and 21 p.i. The HI antibody titers were detected by HI assay. The three H10 viruses were diluted to 4 HAU and then reacted with the chicken or the duck serum, e.g., the BTG/1496/20 was diluted to 4 HAU and then reacted with the chicken and duck serum of the BTG/1496/20 group, respectively.

[b] days post-inoculation.

[c] days post-contact.

[d] The HI antibody titer was negative.

Fig S1 A

PB2

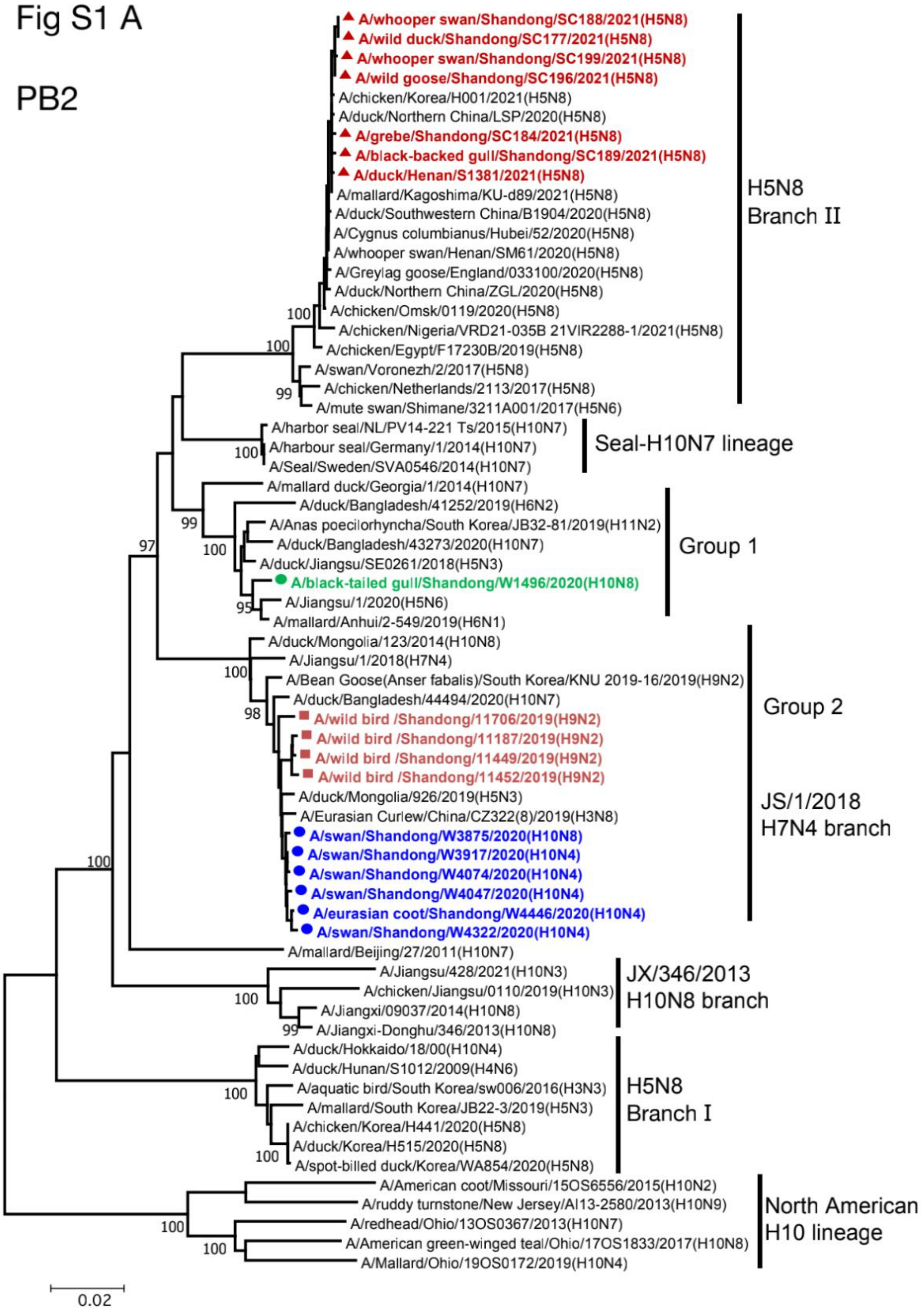
A/whooper swan/Shandong/SC188/2021(H5N8)
A/wild duck/Shandong/SC177/2021(H5N8)
A/whooper swan/Shandong/SC199/2021(H5N8)
A/wild goose/Shandong/SC196/2021(H5N8)
A/chicken/Korea/H001/2021(H5N8)
A/duck/Northern China/LSP/2020(H5N8)
A/grebe/Shandong/SC184/2021(H5N8)
A/black-backed gull/Shandong/SC189/2021(H5N8)
A/duck/Henan/S1381/2021(H5N8)
A/mallard/Kagoshima/KU-d89/2021(H5N8)
A/duck/Southwestern China/B1904/2020(H5N8)
A/Cygnus columbianus/Hubei/52/2020(H5N8)
A/whooper swan/Henan/SM61/2020(H5N8)
A/Greylag goose/England/033100/2020(H5N8)
A/duck/Northern China/ZGL/2020(H5N8)
A/chicken/Omsk/0119/2020(H5N8)
A/chicken/Nigeria/VRD21-035B 21VIR2288-1/2021(H5N8)
A/chicken/Egypt/F17230B/2019(H5N8)
A/swan/Voronezh/2/2017(H5N8)
A/chicken/Netherlands/2113/2017(H5N8)
A/mute swan/Shimane/3211A001/2017(H5N6)
H5N8 Branch II
A/harbor seal/NL/PV14-221 Ts/2015(H10N7)
A/harbour seal/Germany/1/2014(H10N7)
A/Seal/Sweden/SVA0546/2014(H10N7)
Seal-H10N7 lineage
A/mallard duck/Georgia/1/2014(H10N7)
A/duck/Bangladesh/41252/2019(H6N2)
A/Anas poecilorhyncha/South Korea/JB32-81/2019(H11N2)
A/duck/Bangladesh/43273/2020(H10N7)
A/duck/Jiangsu/SE0261/2018(H5N3)
A/black-tailed gull/Shandong/W1496/2020(H10N8)
A/Jiangsu/1/2020(H5N6)
A/mallard/Anhui/2-549/2019(H6N1)
Group 1
A/duck/Mongolia/123/2014(H10N8)
A/Jiangsu/1/2018(H7N4)
A/Bean Goose(Anser fabalis)/South Korea/KNU 2019-16/2019(H9N2)
A/duck/Bangladesh/44494/2020(H10N7)
A/wild bird /Shandong/11706/2019(H9N2)
A/wild bird /Shandong/11187/2019(H9N2)
A/wild bird /Shandong/11449/2019(H9N2)
A/wild bird /Shandong/11452/2019(H9N2)
A/duck/Mongolia/926/2019(H5N3)
A/Eurasian Curlew/China/CZ322(8)/2019(H3N8)
A/swan/Shandong/W3875/2020(H10N8)
A/swan/Shandong/W3917/2020(H10N4)
A/swan/Shandong/W4074/2020(H10N4)
A/swan/Shandong/W4047/2020(H10N4)
A/eurasian coot/Shandong/W4446/2020(H10N4)
A/swan/Shandong/W4322/2020(H10N4)
Group 2
JS/1/2018 H7N4 branch
A/mallard/Beijing/27/2011(H10N7)
A/Jiangsu/428/2021(H10N3)
A/chicken/Jiangsu/0110/2019(H10N3)
A/Jiangxi/09037/2014(H10N8)
A/Jiangxi-Donghu/346/2013(H10N8)
JX/346/2013 H10N8 branch
A/duck/Hokkaido/18/00(H10N4)
A/duck/Hunan/S1012/2009(H4N6)
A/aquatic bird/South Korea/sw006/2016(H3N3)
A/mallard/South Korea/JB22-3/2019(H5N3)
A/chicken/Korea/H441/2020(H5N8)
A/duck/Korea/H515/2020(H5N8)
A/spot-billed duck/Korea/WA854/2020(H5N8)
H5N8 Branch I
A/American coot/Missouri/15OS6556/2015(H10N2)
A/ruddy turnstone/New Jersey/AI13-2580/2013(H10N9)
A/redhead/Ohio/13OS0367/2013(H10N7)
A/American green-winged teal/Ohio/17OS1833/2017(H10N8)
A/Mallard/Ohio/19OS0172/2019(H10N4)
North American H10 lineage
100
100
99
100
99
100
97
95
100
98
100
100
99
100
100
100
100
0.02

Fig S1 B

PB1

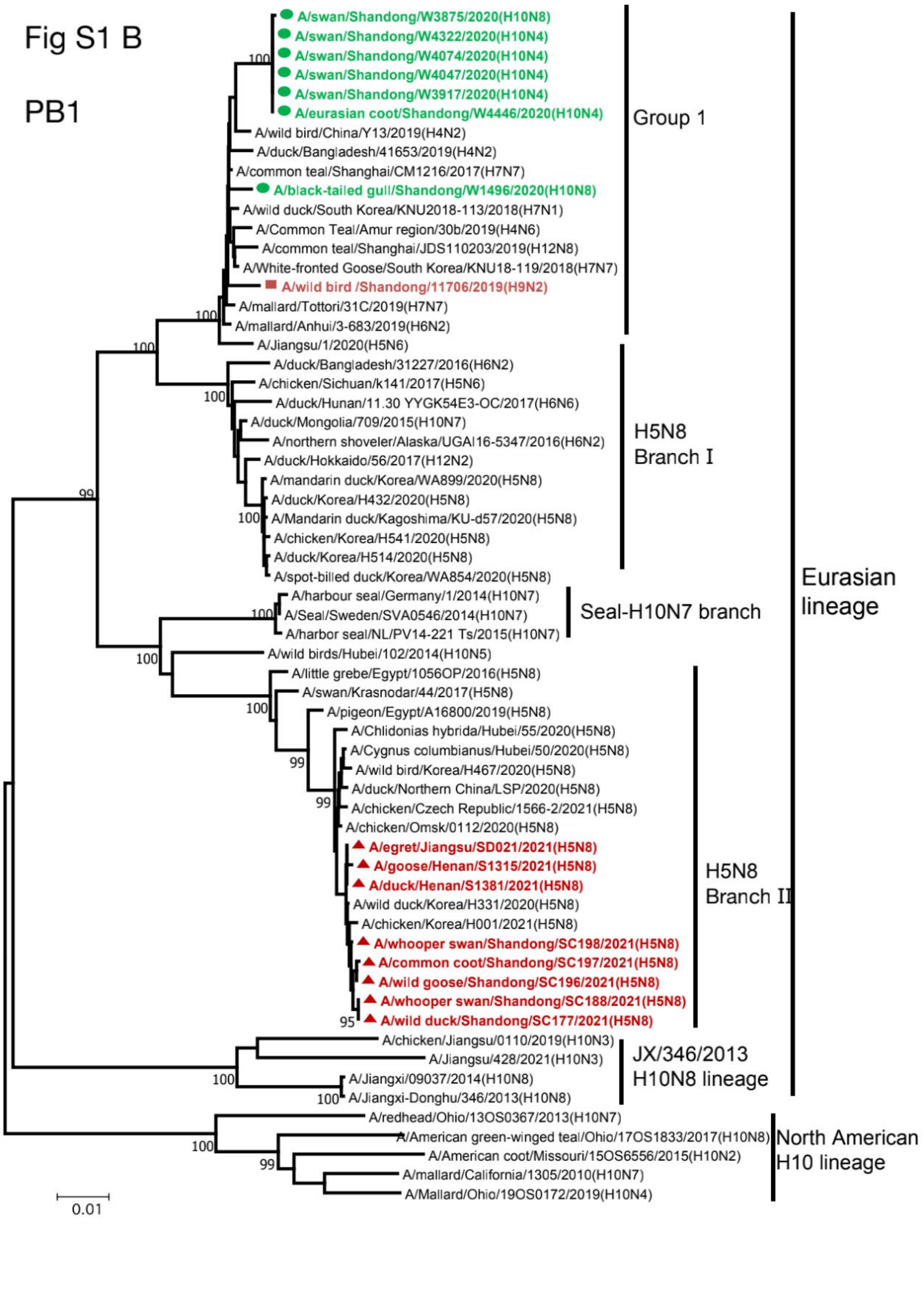
A/swan/Shandong/W3875/2020(H10N8)
A/swan/Shandong/W4322/2020(H10N4)
A/swan/Shandong/W4074/2020(H10N4)
A/swan/Shandong/W4047/2020(H10N4)
A/swan/Shandong/W3917/2020(H10N4)
A/eurasian coot/Shandong/W4446/2020(H10N4)
A/wild bird/China/Y13/2019(H4N2)
A/duck/Bangladesh/41653/2019(H4N2)
A/common teal/Shanghai/CM1216/2017(H7N7)
A/black-tailed gull/Shandong/W1496/2020(H10N8)
A/wild duck/South Korea/KNU2018-113/2018(H7N1)
A/Common Teal/Amur region/30b/2019(H4N6)
A/common teal/Shanghai/JDS110203/2019(H12N8)
A/White-fronted Goose/South Korea/KNU18-119/2018(H7N7)
A/wild bird /Shandong/11706/2019(H9N2)
A/mallard/Tottori/31C/2019(H7N7)
A/mallard/Anhui/3-683/2019(H6N2)
A/Jiangsu/1/2020(H5N6)
Group 1
A/duck/Bangladesh/31227/2016(H6N2)
A/chicken/Sichuan/k141/2017(H5N6)
A/duck/Hunan/11.30 YYGK54E3-OC/2017(H6N6)
A/duck/Mongolia/709/2015(H10N7)
A/northern shoveler/Alaska/UGAI16-5347/2016(H6N2)
A/duck/Hokkaido/56/2017(H12N2)
A/mandarin duck/Korea/WA899/2020(H5N8)
A/duck/Korea/H432/2020(H5N8)
A/Mandarin duck/Kagoshima/KU-d57/2020(H5N8)
A/chicken/Korea/H541/2020(H5N8)
A/duck/Korea/H514/2020(H5N8)
A/spot-billed duck/Korea/WA854/2020(H5N8)
H5N8
Branch I
A/harbour seal/Germany/1/2014(H10N7)
A/Seal/Sweden/SVA0546/2014(H10N7)
A/harbor seal/NL/PV14-221 Ts/2015(H10N7)
Seal-H10N7 branch
A/wild birds/Hubei/102/2014(H10N5)
A/little grebe/Egypt/1056OP/2016(H5N8)
A/swan/Krasnodar/44/2017(H5N8)
A/pigeon/Egypt/A16800/2019(H5N8)
A/Chlidonias hybrida/Hubei/55/2020(H5N8)
A/Cygnus columbianus/Hubei/50/2020(H5N8)
A/wild bird/Korea/H467/2020(H5N8)
A/duck/Northern China/LSP/2020(H5N8)
A/chicken/Czech Republic/1566-2/2021(H5N8)
A/chicken/Omsk/0112/2020(H5N8)
A/egret/Jiangsu/SD021/2021(H5N8)
A/goose/Henan/S1315/2021(H5N8)
A/duck/Henan/S1381/2021(H5N8)
A/wild duck/Korea/H331/2020(H5N8)
A/chicken/Korea/H001/2021(H5N8)
A/whooper swan/Shandong/SC198/2021(H5N8)
A/common coot/Shandong/SC197/2021(H5N8)
A/wild goose/Shandong/SC196/2021(H5N8)
A/whooper swan/Shandong/SC188/2021(H5N8)
A/wild duck/Shandong/SC177/2021(H5N8)
H5N8
Branch II
A/chicken/Jiangsu/0110/2019(H10N3)
A/Jiangsu/428/2021(H10N3)
A/Jiangxi/09037/2014(H10N8)
A/Jiangxi-Donghu/346/2013(H10N8)
JX/346/2013
H10N8 lineage
Eurasian
lineage
A/redhead/Ohio/13OS0367/2013(H10N7)
A/American green-winged teal/Ohio/17OS1833/2017(H10N8)
A/American coot/Missouri/15OS6556/2015(H10N2)
A/mallard/California/1305/2010(H10N7)
A/Mallard/Ohio/19OS0172/2019(H10N4)
North American
H10 lineage
100
100
100
99
100
100
100
100
99
99
95
100
100
100
99
0.01

Fig S1 C

PA

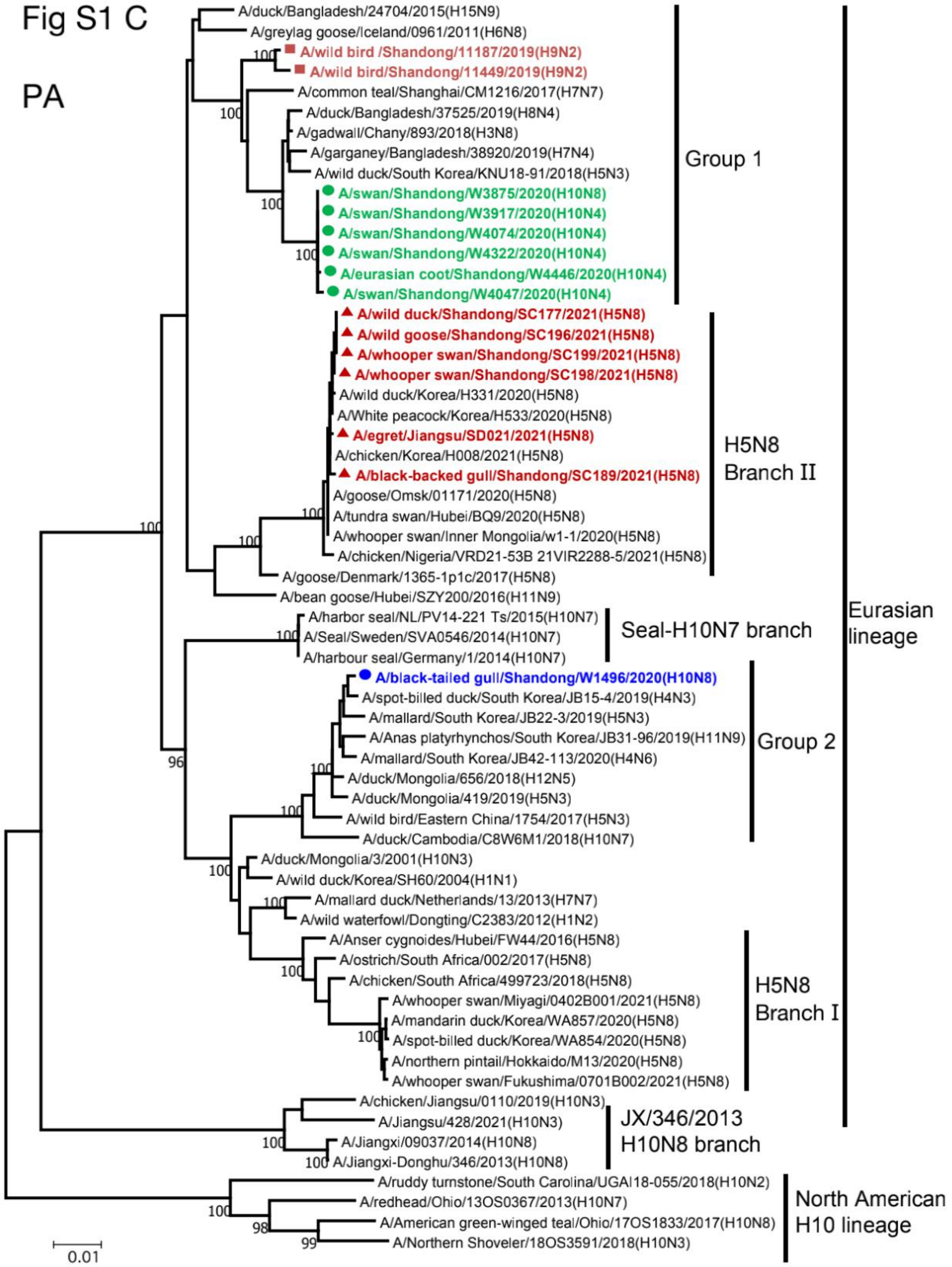
A/duck/Bangladesh/24704/2015(H15N9)
A/greylag goose/Iceland/0961/2011(H6N8)
A/wild bird /Shandong/11187/2019(H9N2)
A/wild bird/Shandong/11449/2019(H9N2)
A/common teal/Shanghai/CM1216/2017(H7N7)
A/duck/Bangladesh/37525/2019(H8N4)
A/gadwall/Chany/893/2018(H3N8)
A/garganey/Bangladesh/38920/2019(H7N4)
A/wild duck/South Korea/KNU18-91/2018(H5N3)
A/swan/Shandong/W3875/2020(H10N8)
A/swan/Shandong/W3917/2020(H10N4)
A/swan/Shandong/W4074/2020(H10N4)
A/swan/Shandong/W4322/2020(H10N4)
A/eurasian coot/Shandong/W4446/2020(H10N4)
A/swan/Shandong/W4047/2020(H10N4)
Group 1
A/wild duck/Shandong/SC177/2021(H5N8)
A/wild goose/Shandong/SC196/2021(H5N8)
A/whooper swan/Shandong/SC199/2021(H5N8)
A/whooper swan/Shandong/SC198/2021(H5N8)
A/wild duck/Korea/H331/2020(H5N8)
A/White peacock/Korea/H533/2020(H5N8)
A/egret/Jiangsu/SD021/2021(H5N8)
A/chicken/Korea/H008/2021(H5N8)
A/black-backed gull/Shandong/SC189/2021(H5N8)
A/goose/Omsk/01171/2020(H5N8)
A/tundra swan/Hubei/BQ9/2020(H5N8)
A/whooper swan/Inner Mongolia/w1-1/2020(H5N8)
A/chicken/Nigeria/VRD21-53B 21VIR2288-5/2021(H5N8)
A/goose/Denmark/1365-1p1c/2017(H5N8)
A/bean goose/Hubei/SZY200/2016(H11N9)
H5N8
Branch II
A/harbor seal/NL/PV14-221 Ts/2015(H10N7)
A/Seal/Sweden/SVA0546/2014(H10N7)
A/harbour seal/Germany/1/2014(H10N7)
Seal-H10N7 branch
A/black-tailed gull/Shandong/W1496/2020(H10N8)
A/spot-billed duck/South Korea/JB15-4/2019(H4N3)
A/mallard/South Korea/JB22-3/2019(H5N3)
A/Anas platyrhynchos/South Korea/JB31-96/2019(H11N9)
A/mallard/South Korea/JB42-113/2020(H4N6)
A/duck/Mongolia/656/2018(H12N5)
A/duck/Mongolia/419/2019(H5N3)
A/wild bird/Eastern China/1754/2017(H5N3)
A/duck/Cambodia/C8W6M1/2018(H10N7)
Group 2
A/duck/Mongolia/3/2001(H10N3)
A/wild duck/Korea/SH60/2004(H1N1)
A/mallard duck/Netherlands/13/2013(H7N7)
A/wild waterfowl/Dongting/C2383/2012(H1N2)
A/Anser cygnoides/Hubei/FW44/2016(H5N8)
A/ostrich/South Africa/002/2017(H5N8)
A/chicken/South Africa/499723/2018(H5N8)
A/whooper swan/Miyagi/0402B001/2021(H5N8)
A/mandarin duck/Korea/WA857/2020(H5N8)
A/spot-billed duck/Korea/WA854/2020(H5N8)
A/northern pintail/Hokkaido/M13/2020(H5N8)
A/whooper swan/Fukushima/0701B002/2021(H5N8)
H5N8
Branch I
Eurasian
lineage
A/chicken/Jiangsu/0110/2019(H10N3)
A/Jiangsu/428/2021(H10N3)
A/Jiangxi/09037/2014(H10N8)
A/Jiangxi-Donghu/346/2013(H10N8)
JX/346/2013
H10N8 branch
A/ruddy turnstone/South Carolina/UGAI18-055/2018(H10N2)
A/redhead/Ohio/13OS0367/2013(H10N7)
A/American green-winged teal/Ohio/17OS1833/2017(H10N8)
A/Northern Shoveler/18OS3591/2018(H10N3)
North American
H10 lineage
100
100
100
100
100
100
100
100
96
100
100
100
100
100
100
100
100
98
99
0.01

Fig S1 D

NP

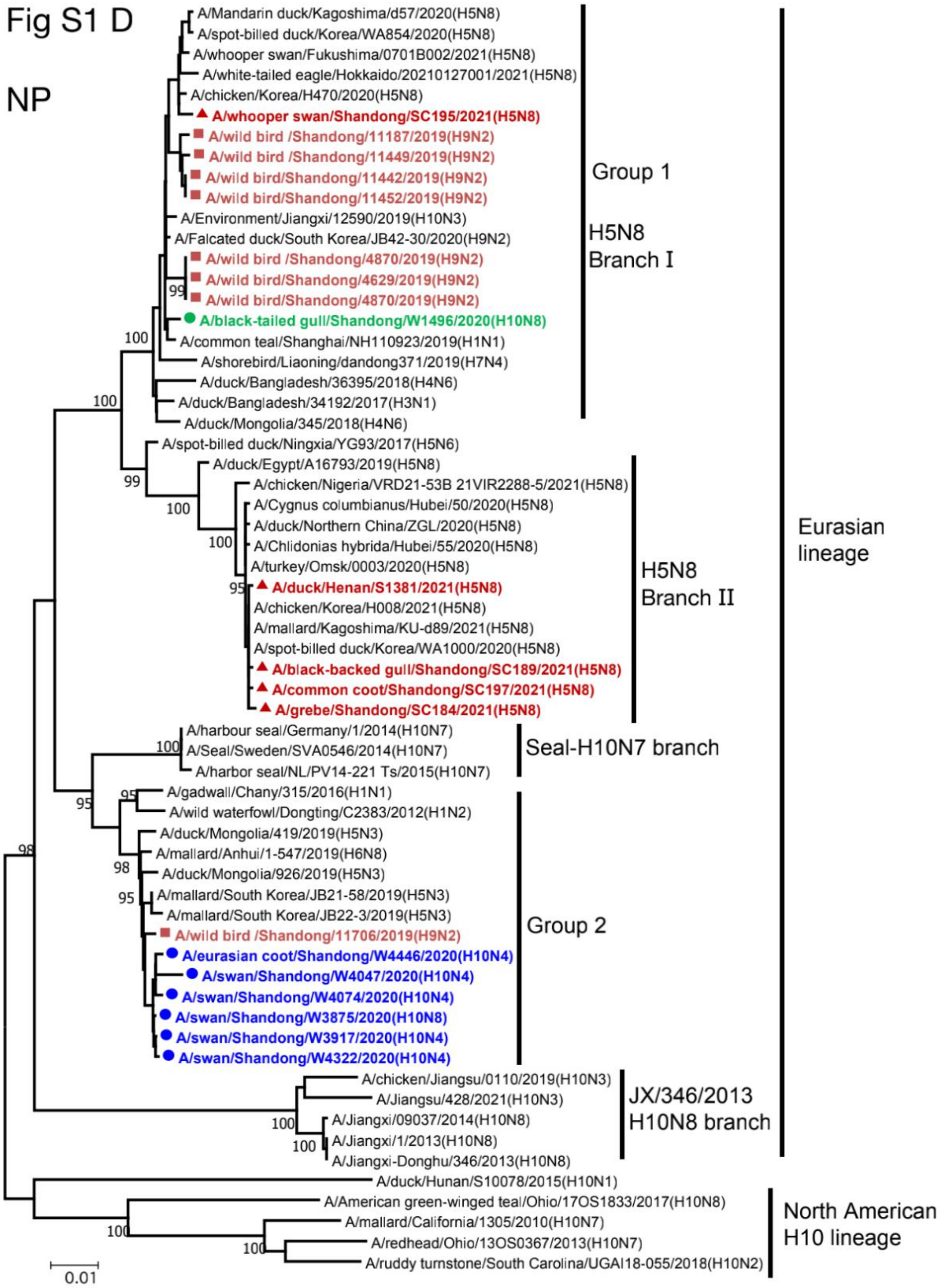
A/Mandarin duck/Kagoshima/d57/2020(H5N8)
A/spot-billed duck/Korea/WA854/2020(H5N8)
A/whooper swan/Fukushima/0701B002/2021(H5N8)
A/white-tailed eagle/Hokkaido/20210127001/2021(H5N8)
A/chicken/Korea/H470/2020(H5N8)
A/whooper swan/Shandong/SC195/2021(H5N8)
A/wild bird /Shandong/11187/2019(H9N2)
A/wild bird /Shandong/11449/2019(H9N2)
A/wild bird/Shandong/11442/2019(H9N2)
A/wild bird/Shandong/11452/2019(H9N2)
A/Environment/Jiangxi/12590/2019(H10N3)
A/Falcated duck/South Korea/JB42-30/2020(H9N2)
A/wild bird /Shandong/4870/2019(H9N2)
A/wild bird/Shandong/4629/2019(H9N2)
A/wild bird/Shandong/4870/2019(H9N2)
A/black-tailed gull/Shandong/W1496/2020(H10N8)
A/common teal/Shanghai/NH110923/2019(H1N1)
A/shorebird/Liaoning/dandong371/2019(H7N4)
A/duck/Bangladesh/36395/2018(H4N6)
A/duck/Bangladesh/34192/2017(H3N1)
A/duck/Mongolia/345/2018(H4N6)
A/spot-billed duck/Ningxia/YG93/2017(H5N6)
A/duck/Egypt/A16793/2019(H5N8)
A/chicken/Nigeria/VRD21-53B 21VIR2288-5/2021(H5N8)
A/Cygnus columbianus/Hubei/50/2020(H5N8)
A/duck/Northern China/ZGL/2020(H5N8)
A/Chlidonias hybrida/Hubei/55/2020(H5N8)
A/turkey/Omsk/0003/2020(H5N8)
A/duck/Henan/S1381/2021(H5N8)
A/chicken/Korea/H008/2021(H5N8)
A/mallard/Kagoshima/KU-d89/2021(H5N8)
A/spot-billed duck/Korea/WA1000/2020(H5N8)
A/black-backed gull/Shandong/SC189/2021(H5N8)
A/common coot/Shandong/SC197/2021(H5N8)
A/grebe/Shandong/SC184/2021(H5N8)
A/harbour seal/Germany/1/2014(H10N7)
A/Seal/Sweden/SVA0546/2014(H10N7)
A/harbor seal/NL/PV14-221 Ts/2015(H10N7)
A/gadwall/Chany/315/2016(H1N1)
A/wild waterfowl/Dongting/C2383/2012(H1N2)
A/duck/Mongolia/419/2019(H5N3)
A/mallard/Anhui/1-547/2019(H6N8)
A/duck/Mongolia/926/2019(H5N3)
A/mallard/South Korea/JB21-58/2019(H5N3)
A/mallard/South Korea/JB22-3/2019(H5N3)
A/wild bird /Shandong/11706/2019(H9N2)
A/eurasian coot/Shandong/W4446/2020(H10N4)
A/swan/Shandong/W4047/2020(H10N4)
A/swan/Shandong/W4074/2020(H10N4)
A/swan/Shandong/W3875/2020(H10N8)
A/swan/Shandong/W3917/2020(H10N4)
A/swan/Shandong/W4322/2020(H10N4)
A/chicken/Jiangsu/0110/2019(H10N3)
A/Jiangsu/428/2021(H10N3)
A/Jiangxi/09037/2014(H10N8)
A/Jiangxi/1/2013(H10N8)
A/Jiangxi-Donghu/346/2013(H10N8)
A/duck/Hunan/S10078/2015(H10N1)
A/American green-winged teal/Ohio/17OS1833/2017(H10N8)
A/mallard/California/1305/2010(H10N7)
A/redhead/Ohio/13OS0367/2013(H10N7)
A/ruddy turnstone/South Carolina/UGAI18-055/2018(H10N2)
100
100
99
99
100
100
95
100
95
95
98
98
95
100
100
100
100
Group 1
H5N8
Branch I
H5N8
Branch II
Seal-H10N7 branch
Group 2
JX/346/2013
H10N8 branch
Eurasian
lineage
North American
H10 lineage
0.01

Fig S1 E

M

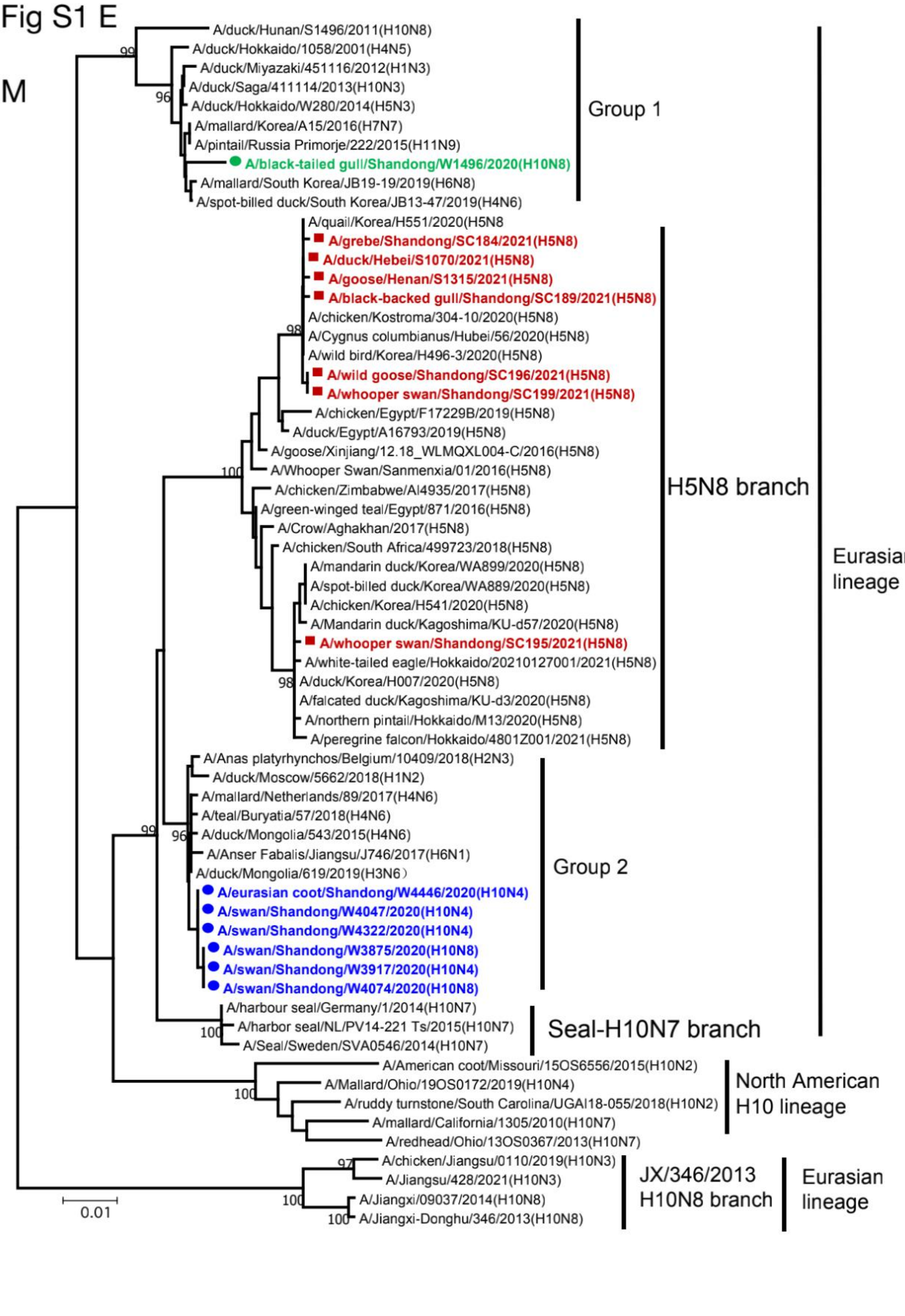
A/duck/Hunan/S1496/2011(H10N8)
A/duck/Hokkaido/1058/2001(H4N5)
A/duck/Miyazaki/451116/2012(H1N3)
A/duck/Saga/411114/2013(H10N3)
A/duck/Hokkaido/W280/2014(H5N3)
A/mallard/Korea/A15/2016(H7N7)
A/pintail/Russia Primorje/222/2015(H11N9)
A/black-tailed gull/Shandong/W1496/2020(H10N8)
A/mallard/South Korea/JB19-19/2019(H6N8)
A/spot-billed duck/South Korea/JB13-47/2019(H4N6)
Group 1
A/quail/Korea/H551/2020(H5N8
A/grebe/Shandong/SC184/2021(H5N8)
A/duck/Hebei/S1070/2021(H5N8)
A/goose/Henan/S1315/2021(H5N8)
A/black-backed gull/Shandong/SC189/2021(H5N8)
A/chicken/Kostroma/304-10/2020(H5N8)
A/Cygnus columbianus/Hubei/56/2020(H5N8)
A/wild bird/Korea/H496-3/2020(H5N8)
A/wild goose/Shandong/SC196/2021(H5N8)
A/whooper swan/Shandong/SC199/2021(H5N8)
A/chicken/Egypt/F17229B/2019(H5N8)
A/duck/Egypt/A16793/2019(H5N8)
A/goose/Xinjiang/12.18_WLMQXL004-C/2016(H5N8)
A/Whooper Swan/Sanmenxia/01/2016(H5N8)
A/chicken/Zimbabwe/AI4935/2017(H5N8)
A/green-winged teal/Egypt/871/2016(H5N8)
A/Crow/Aghakhan/2017(H5N8)
A/chicken/South Africa/499723/2018(H5N8)
A/mandarin duck/Korea/WA899/2020(H5N8)
A/spot-billed duck/Korea/WA889/2020(H5N8)
A/chicken/Korea/H541/2020(H5N8)
A/Mandarin duck/Kagoshima/KU-d57/2020(H5N8)
A/whooper swan/Shandong/SC195/2021(H5N8)
A/white-tailed eagle/Hokkaido/20210127001/2021(H5N8)
A/duck/Korea/H007/2020(H5N8)
A/falcated duck/Kagoshima/KU-d3/2020(H5N8)
A/northern pintail/Hokkaido/M13/2020(H5N8)
A/peregrine falcon/Hokkaido/4801Z001/2021(H5N8)
H5N8 branch
A/Anas platyrhynchos/Belgium/10409/2018(H2N3)
A/duck/Moscow/5662/2018(H1N2)
A/mallard/Netherlands/89/2017(H4N6)
A/teal/Buryatia/57/2018(H4N6)
A/duck/Mongolia/543/2015(H4N6)
A/Anser Fabalis/Jiangsu/J746/2017(H6N1)
A/duck/Mongolia/619/2019(H3N6)
A/eurasian coot/Shandong/W4446/2020(H10N4)
A/swan/Shandong/W4047/2020(H10N4)
A/swan/Shandong/W4322/2020(H10N4)
A/swan/Shandong/W3875/2020(H10N8)
A/swan/Shandong/W3917/2020(H10N4)
A/swan/Shandong/W4074/2020(H10N8)
Group 2
A/harbour seal/Germany/1/2014(H10N7)
A/harbor seal/NL/PV14-221 Ts/2015(H10N7)
A/Seal/Sweden/SVA0546/2014(H10N7)
Seal-H10N7 branch
Eurasian lineage
A/American coot/Missouri/15OS6556/2015(H10N2)
A/Mallard/Ohio/19OS0172/2019(H10N4)
A/ruddy turnstone/South Carolina/UGAI18-055/2018(H10N2)
A/mallard/California/1305/2010(H10N7)
A/redhead/Ohio/13OS0367/2013(H10N7)
North American H10 lineage
A/chicken/Jiangsu/0110/2019(H10N3)
A/Jiangsu/428/2021(H10N3)
A/Jiangxi/09037/2014(H10N8)
A/Jiangxi-Donghu/346/2013(H10N8)
JX/346/2013 H10N8 branch
Eurasian lineage
0.01
99
96
98
100
98
99
96
100
100
97
100
100

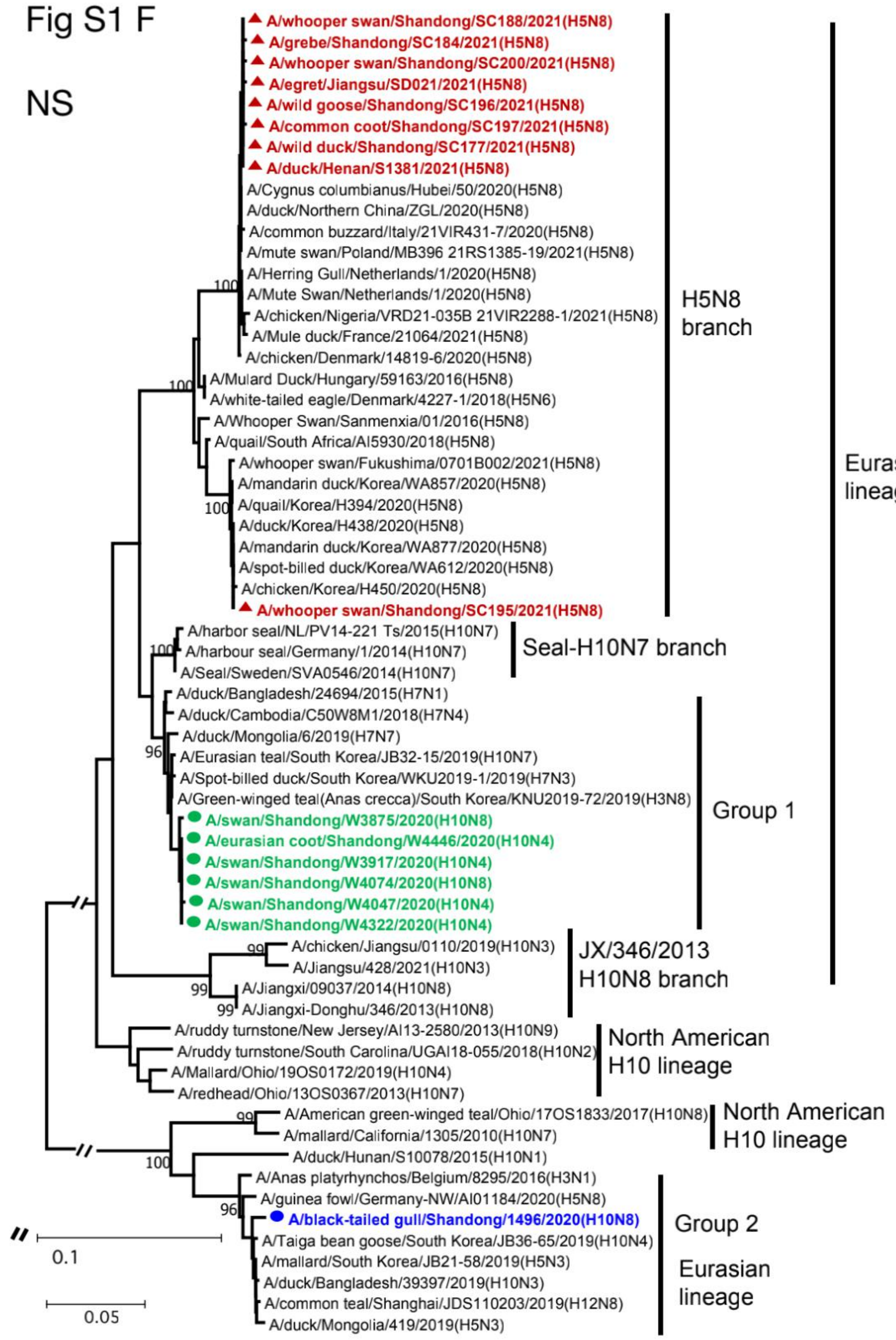

Figure S1. Phylogenetic diagram of PB2 (A), PB1 (B), PA (C), NP (D), M (E), and NS (F) genes of H10N4, and H10N8 viruses. The phylogenetic tree of the six internal genes were constructed by MEGA 7.0 with Neighbor-Joining method. The sequence name colored in green and blue were the viruses isolated in this study, and the sequences name in red were the H5N8 or H9N2 viruses isolated in our previous study. The sequence name with black were downloaded from the database.

Figure S2

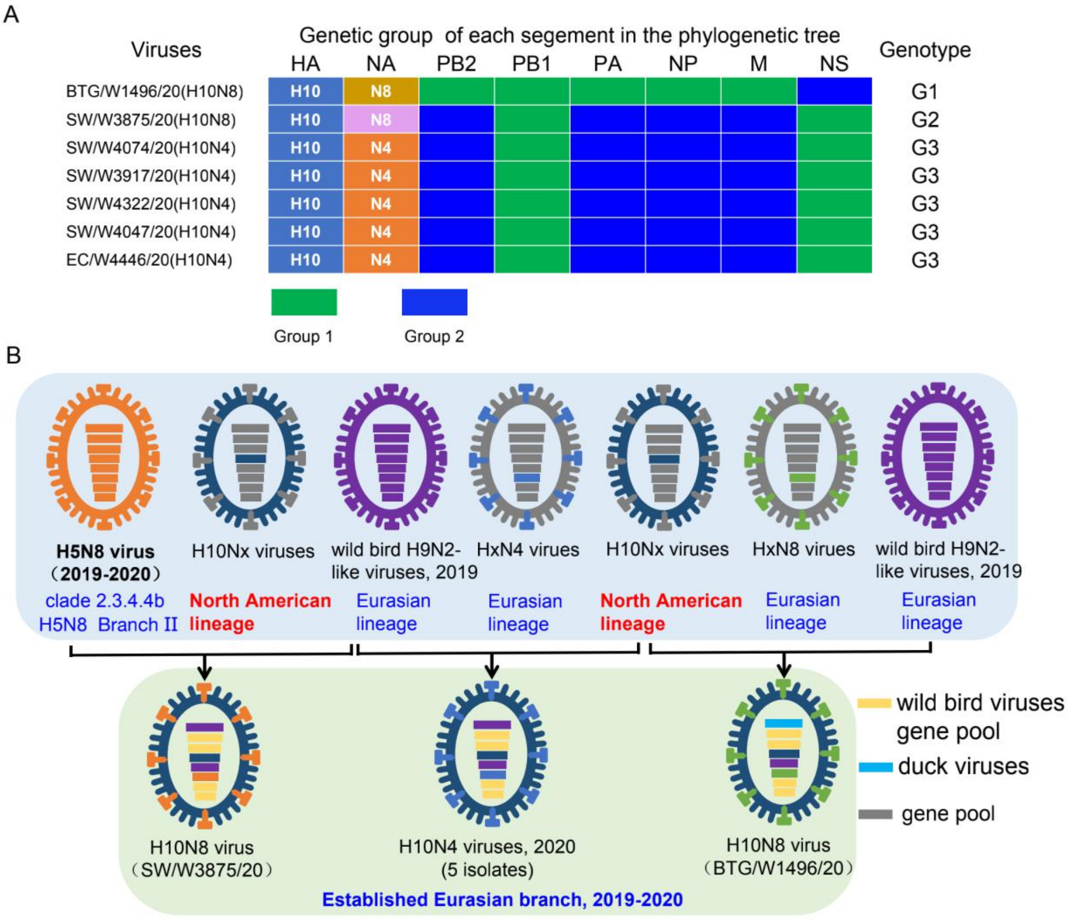

Figure S2. Genotypes and reassortment of H10N4 and H10N8 viruses. (A) Genotypes of H10N4 and H10N8 viruses. Each gene segment of the virus was divided into different groups according to their genetic divergence in the phylogenetic tree. (B) Reassortment of H10N4 and H10N8 viruses in wild birds.

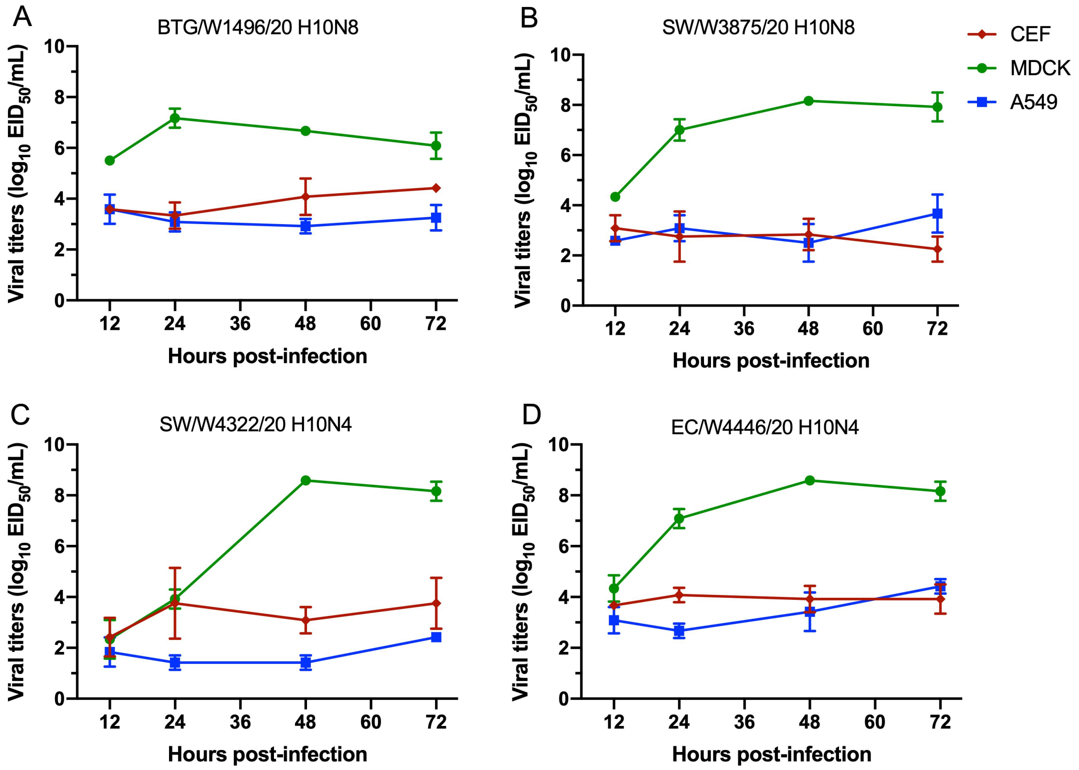

Figure S3. Growth kinetics of H10N4 and H10N8 viruses in chicken and mammal cells. Virus growth curves in MDCK, A549, and CEF cells. The dashed lines indicate the lower limit of detection. Data shown are mean ± SD for three independent experiments, and significance was assessed with a two-tailed unpaired Student's t-test.
